# Computerized text and voice analysis of patients with chronic schizophrenia in art therapy

**DOI:** 10.1038/s41598-023-43069-y

**Published:** 2023-09-25

**Authors:** Yvonne Sprotte

**Affiliations:** Art Therapy Department, Dresden University of Fine Arts (Hochschule für Bildende Künste Dresden), Dresden, Germany

**Keywords:** Diseases, Psychiatric disorders, Schizophrenia

## Abstract

This explorative study of patients with chronic schizophrenia aimed to clarify whether group art therapy followed by a therapist-guided picture review could influence patients’ communication behaviour. Data on voice and speech characteristics were obtained via objective technological instruments, and these characteristics were selected as indicators of communication behaviour. Seven patients were recruited to participate in weekly group art therapy over a period of 6 months. Three days after each group meeting, they talked about their last picture during a standardized interview that was digitally recorded. The audio recordings were evaluated using validated computer-assisted procedures, the transcribed texts were evaluated using the German version of the LIWC2015 program, and the voice recordings were evaluated using the audio analysis software VocEmoApI*.* The dual methodological approach was intended to form an internal control of the study results. An exploratory factor analysis of the complete sets of output parameters was carried out with the expectation of obtaining typical speech and voice characteristics that map barriers to communication in patients with schizophrenia. The parameters of both methods were thus processed into five factors each, i.e., into a quantitative digitized classification of the texts and voices. The factor scores were subjected to a linear regression analysis to capture possible process-related changes. Most patients continued to participate in the study. This resulted in high-quality datasets for statistical analysis. To answer the study question, two results were summarized: First, text analysis factor called *Presence* proved to be a potential surrogate parameter for positive language development. Second, quantitative changes in vocal emotional factors were detected, demonstrating differentiated activation patterns of emotions. These results can be interpreted as an expression of a cathartic healing process. The methods presented in this study make a potentially significant contribution to quantitative research into the effectiveness and mode of action of art therapy.

## Introduction

The discovery and promotion of creative activities for psychiatric patients is considered a milestone in the history of art therapy. Von Spreti and Martius even refer to their stays in “lunatic asylums” in the nineteenth and twentieth centuries as their creative origins^1^. In addition to the unprecedented Heidelberg collection of the art historian and psychiatrist Prinzhorn^2^, the psychiatrist Navratil was one of the first to incorporate pictorial material into psychiatric diagnosis and therapy^3^. The resulting revaluation of individuals with mental illness as artistic-aesthetic creators led to the (hitherto) unknown acceptance of such works by the public^4^.

Regardless of the fascination of many people with the creative work of psychiatric patients, schizophrenia is a serious illness that affects a person as a whole and is accompanied by an incredible disruption of the sense of self^5^. Reduced activity due to social withdrawal and communication problems are typical features of the illness^6^ and have traditionally been identified as the core or basic symptoms of the disease^7,8^. In the actual ICD-10 nomenclature (10th version of the International Classification of Diseases and Related Health Problems), these symptoms are referred to as the ‘negative symptoms’ of the disease and indicate the lack of a previously present mental capacity. This deficiency manifests itself, among other things, in thought and speech disorders leading to confusion, speech peculiarities and language difficulties^7,8^. In addition, the clinical picture of schizophrenia is manifested in peculiarities in affectivity, such as flattening of affect, anxiety, and depressive moods.

For the treatment of schizophrenia, the *S3 treatment guideline for schizophrenia*^9^ recommends a “combination therapy”^9^. This provides for an overall treatment plan based on three pillars: pharmacotherapy, psychotherapy, and psychosocial interventions. Psychosocial interventions also include art-based therapies^9^. According to the S3 treatment guidelines for schizophrenia^9^, art therapies include art, music, dance, and movement therapy as well as theatre and drama therapy^9^. Art therapy is thus included in the group of art-based therapies in the guidelines^9,10^. For patients with a clinical diagnosis of schizophrenia, art therapies are used in outpatient clinics, day clinics, full and partial inpatient psychiatric rehabilitation, forensic psychiatry, and integration assistance^10^. The current S3 guideline for schizophrenia^9^ recommends the use of art therapies under the “recommendation grade B”, i.e., art therapy ‘should’ be provided as part of an overall treatment plan “to improve the psychopathological symptomatology”^9^. Art therapy has thus been established as part of the complementary therapy offered in areas of medicine in which the recovery of patients’ psychological resources is of particular importance. However, this recommendation is not yet based on objective evidence of therapeutic effectiveness; rather, it is essentially based on a no less significant resonance among patients and their clinical or social environment.

The quality of previous attempts to scientifically prove the therapeutic effectiveness of art therapy in schizophrenia patients is assessed differently both in the S3 guideline^9,10^ and by the authors of the *National Institute for Health and Clinical Excellence* (*NICE*^11^) guideline. The only consensus is that, based on the studies, the therapeutic potential for art-based therapies and for art therapy tends to lie in influencing negative symptoms^10^. The most recent studies on the effectiveness of art therapy in schizophrenia patients were reported by Ruiz et al. in 2017^12^.

According to Ruiz et al.^12^, these reviews include four *randomized controlled trials* (RCTs) that have been classified as scientifically usable in the sense of *evidence-based medicine* (EBM). These studies tend to show that the use of art therapy can have a positive effect on negative symptomatology in schizophrenia patients^13–16^.

Since these disease characteristics are difficult to influence by medication, art-based therapies offer complementary healing methods. Through nonverbal methods of expression, these therapies provide alternative ways to communicate with patients and then, if necessary, develop a common thread of conversation. This is particularly successful if an art-scientific-phenomenological point of view is taken in the joint viewing and discussion of self-designed pictures^4,17^. Patients may then respond more reliably to questions, speak more fluently, and appear calmer and more relaxed, creating a more coherent conversational situation. With these observations, a patient´s communication behaviour, their speech and voice, their chosen words and word contexts could express their inner psychological processes and thus provide a research tool for art therapy. This train of thought led to the idea of using quantitative computer-assisted text and voice analysis methods to objectify these observations or the processes on which they are based.

### Automated text and voice analysis as a diagnostic tool in schizophrenia research

The automated analyses performed in the present study to evaluate the digital audio files were based on the current versions of the corresponding technologies. In basic psychiatric and psychological research, speech and voice analysis methods have been used for approximately 30 years to objectify the intrapsychic processes and specific phenomena of mental illnesses (e.g.^18–21^). To date, these methods have not been used in art therapy research.

The extraction of text features with automated lexical analysis can produce objective language metrics that offer valuable insight into cognitive, social, and psychological processes^22,23^. Among these investigative tools is the *Linguistic Inquiry and Word Count* (LIWC) computer-assisted text analysis program^24^.

The contribution of the LIWC program in providing diagnostic information about the mental health of people with schizophrenia has been shown by recent clinical studies, especially in the English-speaking world. Exemplary studies on expressive writing by Junghaenel et al.^19^ as well as studies on free speech by Minor et al.^21^, Hong et al.^25^, Bonfils et al.^26^ and Just et al.^27^ in German-speaking countries should be mentioned in this context. The latter study showed that the interview length of the groups of patients with and without formal thinking disorders differed in that patients with formal thinking disorders had longer interviews and used more words than the healthy control subjects. In contrast, the interviews length of patients without formal thinking disorders was shorter, and these patients also used fewer words than the control subjects^27^.

In summary, the results of these studies indicate that the LIWC analysis instrument is suitable for providing diagnostically useful information about schizophrenia patients. However, Bonfils et al.^26^ and Junghaenel et al.^19^ also noted that further studies should replicate the results and examine them with additional clinical variables. In the future, LIWC analysis could serve as a basis for objective clinical tests in psychiatry^19,26^. However, the 2001 and 2007 versions of the LIWC program have not been used for schizophrenia patients in the German-speaking world thus far. Only the study by Just et al.^27^ examined language samples from schizophrenia patients with the latest German adaptation of the LIWC program, DE-LIWC2015^28^.

Research findings on automated voice analysis among patients with psychiatric or schizophrenic disorders show commonalities in the selection of study instruments and target parameters.

In their review of 127 studies, Low et al.^22^ examined the use of speech processing technologies for automated assessment in patients with psychiatric disorders^22^. According to this review, acoustic feature extraction was predominantly performed using open-source packages such as openSMILE, covarep, pyAudioAnalysis, openEAR, and PRAAT^22^. The main target parameters in patients with psychiatric disorders include prosodic features of fundamental frequency (F0), variability in fundamental frequency, loudness, speech tempo, variability in loudness, the duration of speech, and acoustic features of jitter and shimmer^22^.

Although the study results for schizophrenia patients are not uniform, commonalities have nevertheless emerged in the automated assessment of negative symptoms. This primarily concerns the symptom of speech (alogia). For this symptom, the acoustic parameters of the total duration of conversation^29,30^ and speech rate are mentioned^18,31–34^, which are lower in schizophrenia patients than in control subjects. In contrast, studies have shown that the average pause duration indicates higher measured values^18,29,30,33–36^. Second, this relates to flat affect as another feature of negative symptomatology, expressed by both a lower baseline frequency (F0) and lower baseline frequency variability in schizophrenia patients^33,34,36^.

In addition to analysing speech and voice alone, there are also studies that combine, for example, the measurement of voice and facial expression features^37,38^. To measure facial expressions, the recent study by Abbas et al.^38^ used the OpenFace software library, whose computer version is based on Ekman and Friesen’s *Facial Action Coding System* (FACS)^39,40^. For prosodic parameters, they also chose the mean fundamental frequency, variability in fundamental frequency and loudness as acoustic features. As the most important result, Abbas et al.^38^ demonstrated that the mean fundamental frequency and loudness correlate negatively with the severity of negative symptomatology^38^.

In summary, the listed studies on voice analysis in schizophrenia patients show that the prosodic features of total speech duration, speech rate, fundamental frequency, and variability in fundamental frequency can be used to map classic symptoms of negative symptomatology, such as poverty of speech and flat affect in schizophrenia patients^23^.

## Methods

### Ethical statement

The art therapy study was approved by the management of the privately run *Marienheim*, a social therapy facility for individuals with mental illness. Written project permission was received on 30 March 2016. The written, informed consent of all seven study participants was also obtained before the start of the study. The legal guardians of these seven patients were also informed about their wards’ study participation and made the consent forms legally binding by signing them. After completion of these formal requirements, the author drew up the final list of study participants, which was signed by the two treating psychiatrists. They endorsed the art therapy envisaged in the study and described it as a ‘medically indicated therapeutic measure’ integrated in the overall treatment plan. Furthermore, the Ethics Committee of the University of Augsburg confirmed the ethical harmlessness of the art therapy research project in its statement dated 11 May 2017. The art therapy study was thus carried out according to the principles of the Declaration of Helsinki.

The study was registered at www.isrctn.com (identifier: ISRCTN12365070; 10.1186/ISRCTN12365070).

### Sampling

Seven patients (n = 7) aged between 46 and 62 years were included in the explorative single case study (SSR in an isolated B design) over a period of six months (05/2016 to 10/2016). They were inpatients at the *Marienheim* facility in Peiting and had long-term schizophrenia. The patients of the *Marienheim* were people of all genders who had different, partly combined mental illnesses classified using ICD codes. Their stay was under a judicial accommodation order according to §1906 BGB, and they had legal care. The construction of the sample from the total population for this art therapy study resulted primarily from the specific objective. Second, the complete lack of third-party funding for this research project also played a role, albeit a subordinate one.

The *Marienheim* was occupied by 69 patients in February 2016 (according to the resident list on 12 February 2016). The selection of the sample took place under the inclusion and exclusion criteria in cooperation with both attending consultant physicians. One physician was a specialist in neurology and psychiatry from an independent practice, and the other was a specialist from a psychiatric outpatient clinic (PIA); they were both commissioned by the *Marienheim* facility.

The inclusion criteria were as follows: a diagnosis of paranoid schizophrenia (F20.0) with chronic course of at least 10 years and/or residual schizophrenia (F20.5), stable medication use for psychotic symptoms for at least 3 months, a preference for art-based therapies (art therapy), and the presence of a written informed consent form. The exclusion criteria were as follows: primary addictive disorder, acute accessory symptoms, suicidality, intelligence reduction, brain organic disorder, autistic disorder, personality disorder, visual impairment, language barrier, hemiparesis including speech disorder, upcoming discharge, or participation in another creative therapy (in group and/or individual therapy).

Based on the above ‘inclusion and exclusion criteria’, 15 patients were determined to be suitable for the sample by the treating psychiatrists on 09 April 2016. Therefore, the author informed the 15 patients about the details of the study in individual interviews, offering them the opportunity to participate. The obligatory ‘information and consent form’ was also discussed in detail and provided if needed. Of these 15 patients, eight agreed to participate in the study. After a reflection period of several days, seven patients signed the ‘information and consent form’. One study participant (P7) asked to participate in the study group but did not allow audio recording of the interviews. The list of study participants, signed by the psychiatrists—not anonymized in the original—is shown in Supplementary Table A.1, including the year of birth, sex, diagnosis(es), and date of admission into the *Marienheim.* In the further course of the study, the study patients were named exclusively as P1, P2, P3, P4, P5, P6, (P7), and P8.

### Procedure

**Figure 1:**
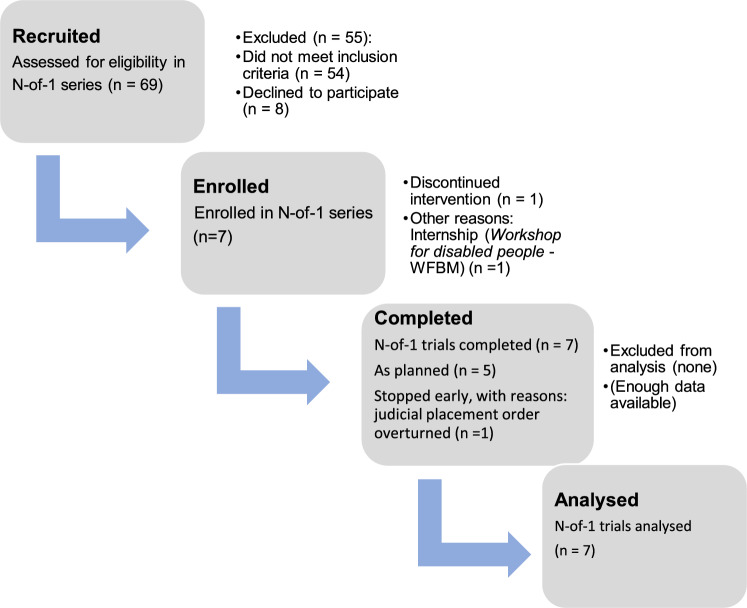
CENT flow diagram; suggested representation of the flow of participants in a series of N-of-1 trials 10.1136/bmj.h1738^41^.

The course of the individual study phases with specifics of study interruptions/dropouts with reasons are shown in the CENT-2015 Flow Diagram in Fig. 1.

### Art therapy intervention

#### Picture creation

Group art therapy with image creation took place once a week, always on Fridays, from 10:00 am to 11:30 am over a period of 6 months (05/2016 to 10/2016), with up to 20 possible sessions.

The author moderated through the three phases, consisting of the opening, picture design and closing rounds, according to Schrode^42^ and von Spreti^43^. In the opening round, which lasted approximately 15 min, each participant was given the opportunity to talk about how they were currently feeling. Especially at the beginning of the art therapy study—when the participants did not know each other very well—it was the author’s task to create a trusting atmosphere within the art therapy group^43^. This was followed by the author setting the tasks and themes for the day to create the pictures. The tasks and themes were a central element in the art therapy study approach. The empirical findings of the art therapist Landgarten^44^ formed the basis for the tasks and themes. The detailed description of the tasks and themes for the creation of the pictures is described in detail in Supplementary Information A.2.

#### Picture reflection

*Therapist-Guided Picture Reflection* (TGPR) took the form of a guided interview questions. Although guided interviews are a “semistructured forms of data collection for obtaining verbal data”^45^, they were used as a structured form of data collection in this research. This was because the questions in the guide enabled the necessary structure in the sense of a standardized survey course. At the same time, this interview form allowed enough space for ‘free speech’, which was required here to obtain sufficiently large speech and voice samples. Furthermore, the standardization of the interview procedure established a certain comparability of the interviews during the study, specifically for intraindividual comparisons of each participant’s speech and voice over the course of six months and for interindividual comparisons across the study group. The TGPR structure (see Table A.3 under Supplementary Information) was based on four phases according to Misoch^45^: the “information phase”, the “warm-up and introduction phase”, the “main phase”, and the “fade-out and conclusion phase”^45^.

It was necessary to create a catalogue of questions (see Table A.4 under Supplementary Information) that, despite stereotypes, allowed for the possibility to create the image in the same way as the essential emotional arc of tension, which was the actual therapeutic moment of the interview.

The thematic areas of the question catalogue were based on the art-therapeutic method of picture discussion from a phenomenological perspective according to Betensky^17^ and the art-based approaches to picture viewing and discussion according to Stuhler-Bauer and Elbing^46^, Bader^47^, Dannecker^4^ and Titze^48^, and according to the specially elaborated conversational style for psychotherapy with schizophrenia patients according to Süllwold^49,50^. In the interviews for data collection, the study participants talked about the images they had created. The conversation about each study patient’s own pictures took place on Monday or Tuesday afternoon in the week after the pic as individual therapy in the form of an interview with a maximum duration of 50 min.

### Research instruments

This art therapy study aimed to record the vocal expression of emotion or emotionality and the speech and thinking style from the verbal and paraverbal speech signals of the study participants. To this end, the study used two computer-assisted research methods to analyse digitized audio recordings, the computer-assisted quantitative text analysis program *Linguistic Inquiry and Word Count* (LIWC2015) and the voice analysis software *Vocal Emotion recognition by Appraisal Inference*, (VocEmoApI technology). Thus, a dual-automated, computerized analysis of the audio documents was achieved.

#### Range of the target parameters of the LIWC2015 program

The study interviews were conducted in German. To evaluate the recordings, the current software version of the LIWC2015 program according to Pennebaker et al.^51^ was combined with a working version of the German LIWC2015 program by Meier et al.^28^. The analysis took place in 2017/2018. The German DE-LIWC2015 dictionary consists of approximately 18,000 words and word stems^28^. In the same way as in the English version of the LIWC2015 program, the recognized words can be assigned to several word categories^28^. The quality of the German LIWC2015 program was evaluated by Meier et al.^28^ in two studies: first, by comparing the German translation (DE-LIWC2015) with the original English translation (ENG-LIWC2015) and then with the previous German version, the DE-LIWC2001 program. The findings in the studies showed a high level of equivalence between the two dictionaries^28^.

The computer-assisted text analysis LIWC program is a computer program that automatically examines written or transcribed verbal texts for features determined by the author in a formal, quantitative way. In doing so, the application focuses less on the content of human speech and more on individual words and word stems, which are counted, assigned to defined word categories and illustrated as a percentage in relation to the text length^52^. The recognized words can be assigned to several word categories—the so-called upper and lower categories—at the same time and consequently be recorded and counted several times. For example, the word “*cried”* can therefore be assigned to the categories *sad* (sadness), *affect* (all affect words as an upper category), *negemo* (negative emotion), *verb* (verbs), and *focus past* (past)^51^.

For the recording and automatic evaluation of the verbal speech signals, a complete transcription of all individual interviews (full transcription) was necessary. The transcription rules were those from the LIWC2015 user manual according to Pennebaker^51^ and the “German LIWC” program according to Meier et al.^28^.

The LIWC2015 text analysis consists of 90 output parameters (see Supplementary Information under A.5). For the present work, however, only 61 of these parameters were included, since the remaining 29 may not be included in the statistics as an additional share of an upper- or lower-level LIWC2015 category.

For the statistical analysis, how high the percentage of speech data was in an audio document was relevant. For this purpose, the LIWC output parameter Dictionary Words (*Dic*) was used, which determines the proportion of words that are recorded and evaluated by the LIWC2015 text analysis procedure. In addition, it was necessary to select the study-relevant parameter sets for the exploratory factor analysis. These included the three general descriptive variables with the words counted per interview (*WC*), the percentage of sentence length (*WPS*) and the number of words longer than six characters (*Sixltr*). Of the output parameters for the categories ‘basic linguistic and grammatical parameters’ (I) and ‘psychological processes’ (II), only those belonging to a subordinate category were included in the exploratory factor analysis. Therefore, of the ‘basic linguistic and grammatical parameters’ (I), all subcategories with 18 language features were included. This concerned the five linguistic features of personal pronouns (*I*, *we*, *you*, *shehe* and *they*) and impersonal pronouns (*ipron*) as well as the linguistic categories “article” (*article*), “prepositions” (*prep*), “auxiliary verbs” (*auxverb*), “adverbs” (*adverb*), “conjunction” (*conj*) and “negation” (*negate*), “verbs” (*verb*), “adjectives” (*adj*), “comparative words” (*compare*), “interrogative words” (*interrog*), “number words” (*number*) and “words that quantify” (*quant*). Additionally, of the word categories for thematic content ‘psychological processes’ (II), only the subcategories with 30 parameters were included in the exploratory factor analysis. These included two parameters relating to affective processes (*posemo*, *negemo*), four relating to social processes (*family, friend, female, and male*), six relating to cognitive processes (*insight*, *cause*, *discrep*, *tentat*, *certain*, and *differ*), three relating to perception (*see*, *hear*, and *feel*), and four relating to biological processes (*body*, *health*, *sexual* and *ingest*). In addition, five parameters describing drives (*affiliation*, *achieve*, *power*, *reward*, and *risk*), three describing temporal orientation (*focuspast*, *focuspresent*, and *focusfuture*), and three describing relativities (*motion*, *space*, and *time*) were analysed. Finally, six parameters for ‘personal concerns’ (*work*, *leisure*, *home*, *money*, *relig*, and *death*) and four for ‘formless language’ (*swear*, *assent*, *nonflu*, and *filler*) were included*.* In summary, 61 parameters from the LIWC2015 text analysis were included in the exploratory factor analysis.

#### Range of target parameters of VocEmoApI

The voice analysis procedure used here was the further development of a software called sensAI (*sensitive Audio Intelligence*), a technology for the computer-aided identification of affective speaker states via human speech or voice. The following description of the software based on the working manual “sensAI WebAPI Documentation—Version 1.3 from 5.12.17” was used^53^. SensAI technology analyses the input audio files and scales the automated evaluation of the output variables from speech or voice as well as emotional content. It recognizes the human voice with the help of “voice activity detection” (VAD) and can also distinguish human voices from loud background noises (e.g., street noise). The speech signals of the speaker are decoded into characteristic and personal features (personality, age, and gender), patient emotional speech states (emotional categories such as joy, anger, and fear), the speech activity (duration, speech tempo, etc.), and the speech prosody (volume, pitch of voice, etc.).

The software, which works with 88 parameters, is based on the basic standard parameter set *Geneva Minimalistic Acoustic Parameter Set* (GeMAPS) for voice research and affective computing^54^. It was applied in the voice analysis of the present study under the name VocEmoApI technology with category_v2_scores (see Supplementary Information under A.6). This includes the intensity parameter, 52 emotion scores for the category_v2_scores, the three emotion dimensions *pleasantness*, *urgency,* and *control*, and the four prosodic features *pitchAverage*, *pitchVariation*, *loudnessAverage* and *speakingSpeed*, which will be explained in the following paragraph. The parameter *intensity* (Line 4) is used to check the emotional intensity of the category_v2_scores. The range is from 0.0 (neutral) to 1.0 (*highly emotional*). A score below 0.25 can be considered neutral language. Scores above this indicate *emotionally coloured* speech^53^.

The 52 emotion scores are found in the category_v2_scores (Line 5). For category_v2_scores, a score reference range is given. This reference range was determined from voice samples of unspecified speakers. It is set between the values − 1.0 and + 1.0. Scores between − 1.0 and 0 are called poor matches, i.e., ranging from the recognized emotion ‘does not fit at all’ (− 1.0) to fits ‘insignificantly’ (0) into the designated category^53^. Values between 0 and 1.0 indicate a good match and quantify the gradual fit of the acoustic signal into the specific emotion category. A value of 1.0 cannot be exceeded in this definition of the reference range; here, the recognized emotional signal fits completely into the designated category. All 52 category_v2_ emotion scores were included in the explorative factor analysis. The three emotion dimensions *pleasantness*, *urgency* and *control* are in Line 6. The scores of *pleasantness* are between − 1.0 (negative), 0.0 (neutral/in between) and 1.0 (positive). The scores of *urgencies* and *control* are between − 1.0 (low), 0.0 (normal), and 1.0 (high)^55^. The four prosodic features *pitchAverage* (average fundamental frequency F0 in Hz), *pitchVariation* (variation of the fundamental frequency F0 in Hz), *loudnessAverage* (average perceived loudness of the speaker) and *speakingSpeed* (speaking tempo in syllables per second) are in Line 8. Pitch is expressed in semitones relative to a base note (A0) of 27.5 Hz. The lowest value for pitch is 12 semitones (55 Hz) and the highest value is 62 semitones (~ 1000 Hz). For loudness, a psychoacoustically corrected loudness measure is employed, considering the human ear’s selective frequency response and nonlinear frequency and intensity perception. Speech speed (*speakingSpeed*) also includes a speaker’s pauses. Thus, values below 3–4 Ss indicate slow speaking, and values above 4 Ss indivate fast speaking. Values below 2 Ss result from hesitant speaking, short utterances or when many pauses are made while speaking^53^. From the total dataset of 88 parameters, 28 were left out for further calculation. With the remaining 60 selected parameters, the affective features from the human voice were analysed.

### Statistical analysis

The analysis of all datasets and the graphical visualization of the results were carried out with the *statistics programmed Statistics Standard* from IBM Version 25.0 (SPSS®). The graphs were also created alternatively with the software *GraphPad Prism* version 8.1.0. (221) (GraphPad®) if this resulted in optimized visualization.

The statistical evaluation of the LIWC2015 and VocEmoApI datasets was carried out using descriptive statistics, exploratory factor analysis, and linear regression analysis.

#### Descriptive statistics

First, in the process of data analysis, the mean (*M*), range (*Range)*, minimum (*Min*), maximum (*Max*), and standard deviation (*SD*) were calculated as characteristics of the measured output parameters for both test methods. This was done in tabular form for the entire study group and for each individual study participant.

Regarding the correlation of study outcomes from the dual study instruments used, the effect size of the bivariate correlation coefficient (r) was defined according to Cohen^56^ as ‘small’ for an r greater than/equal to 0.10, as ‘medium’ for an r greater than/equal to 0.30 and ‘large’ for an r greater than/equal to 0.50^57^.

#### Exploratory factor analysis

*Exploratory factor analysis* (EFA) was used to evaluate the extensive output parameters of the LIWC2015 text analysis (with 61 parameters) and the voice analysis category_v2_scores (with 52 parameters). As a prerequisite for exploratory factor analysis, the following statistical procedures or tests were applied to the output parameters of both research methods: 1. A bivariate correlation matrix according to Pearson^58^. The effect size of the correlation coefficient (r) was defined according to Cohen^56^ as ‘small’ for an r greater/equal 0.10, as ‘medium’ for an r greater/equal 0.30 and ‘large’ for an r greater/equal 0.50^57^.

2. As a further prerequisite, the standard test procedure developed by Kaiser, Meyer, and Olkin (KMO) was applied with the so-called KMO value. A KMO value > 0.60^58^ was assumed to be an acceptable lower limit for suitability. Horn’s parallel analysis was used to determine the number of factors^55^. 3. To check the internal consistency of the factors, a reliability test was carried out using Cronbach’s alpha^58^. With the exploratory factor analysis completed, further statistical analysis of the study was based on the factor scores alone.

#### Linear regression analysis

The following criteria are stated as prerequisites for linear regression analysis: first, an interval scaling of the independent and dependent variables; second, a linear relationship between the two variables; third, a ‘random’ sample; fourth, a normal distribution of the residuals; and fifth, a variance of the residuals (homoscedasticity)^59^. Since the method of nonprobabilistic sampling was used in this study with seven study participants (n = 7), the ‘random sampling’ requirement was not met. However, this exploratory study considered all statistical findings exclusively in a chance-critical manner^57^.

The strength of the linear relationship between the *independent variable* (IV) and *dependent variable* (DV) was indicated by the regression coefficient (b). In addition, a ‘sample significance test’ was performed using a t test to test the regression coefficient (b). The probability level of the t value was explained with p < 0.05. The coefficient of determination (R^2^) provided information about the quality of the regression model^57^. For all seven individual cases, the extracted factors of the two study instruments were visualized as a scatter plot with a regression line, including a 95% confidence interval. The individual interpretation of the process courses was based both on the visual examination of the graphical representations for a normal distribution (histogram) and on the interference statistical data for linear regression analysis.

### Ethics declarations

Due to the lack of invasiveness of the study approach, the study supervisory team did not initially consider an ethics vote to be necessary. Thus, a subsequent vote in the sense of ethical clearance was granted on 11/05/2017 by the chairman of the ethics committee of the University of Augsburg (Prof. Dr. Ulrich M. Gassner, Mag. rer. publ. M. Jur. (Oxon.) Faculty of Law, P.O. Box 86135 Augsburg, Germany; +49 (0) 821 598 4600; ulrich.gassner@jura.uni-augsburg.de.

## Results

### Descriptive statistics

#### LIWC2015

The speech recordings of the study participants showed a wide range from a minimum of 87 to a maximum of 1486 counted words (*WC*). On average (mean = M), the seven study participants used 467 words per interview session (*SD* = 247, range 87–1486; see Table 1, Supplementary B.1.1.1). The mean distribution of the LIWC2015 parameter *Dic* (*Dictionary words*) over the entire sample showed that the percentage of words that the programmed recorded on average from all 115 transcribed interviews in this study was 90% (*SD* = 3%, range 82–96%) (see Table 1). However, it should be critically noted that among the remaining 10%, art or art therapy key words (such as words related to the colours ‘blue’ and ‘black’ or the artistic methods ‘collage’ and ‘watercolour’) were not recorded. Although the LIWC2015 text analysis focused less on the thematic references of the speaker than on the syntactic connections of the words in sentences, in some word count analyses, ambiguities in language use were not considered. This became visible in the individual case analysis of P3. In the first interview (09.05.16), P3 showed the highest percentage of negative emotion words (*negemo* = 2%) during the study, at 2%. This was due to a misinterpretation of the LIWC2015 text analysis software, in which the word ‘monkey’ was counted as a negative emotion word, and P3 used this word several times in the description of her collage.

**Table 1:** Mean distribution (*M*) of the word count (*WC*), dictionary words (*Dic*) and the lead parameters for each study participant and the total collective (TC).

LIWC2015	Study participants							
Parameters	P1	P2	P3	P4	P5	P6	P8	TC
	M (SD)	M (SD)	M (SD)	M (SD)	M (SD)	M (SD)	M (SD)	M (SD)
*WC*	409 (309)	645 (278)	401(134)	362 (133)	548 (196)	687 (293)	298 (83)	467 (247)
*Dic*	91 (3)	91 (3)	89 (3)	87 (4)	92 (2)	88 (2)	89 (3)	90 (3)
*Sixltr*	15 (1)	17 (2)	17 (4)	19 (4)	17 (2)	22 (2)	19 (2)	18 (3)
*Ipron*	16 (2)	13 (2)	13 (2)	13 (2)	15 (3)	12 (2)	14 (2)	14 (3)
*Article*	15 (2)	12 (2)	12 (3)	14 (2)	13 (3)	13 (3)	13 (2)	13 (3)
*Conj*	20 (3)	19 (2)	16 (2)	16 (2)	21 (2)	14 (3)	16 (3)	17 (3)
*Verb*	19 (2)	20 (2)	20 (3)	16 (3)	18 (2)	15 (2)	15 (2)	18 (3)

All other output parameters are shown in %.

*M* mean, *SD* standard deviation, *TC* total collective, *WC (word count)* number of words used.

The detection frequency of the 61 parameters from the LIWC2015 machine text analysis was ranked by the mean values (*M*) for each study participant and across all interviews. A participant’s personal set of guiding parameters was created from the five highest-ranking parameters) (see Table 1, Supplementary B.1.1.1). As a result, the same guiding parameters were determined for the seven study participants and were similarly distributed across the entire group. All seven study participants used a comparable number of words of the following characteristics: “verbs” (*verb*) (*M* = 18%, *SD* = 3%, *range* 11–25%), “words in the subjunctive” (*conj*) (*M* = 17%, *SD* = 3%, *range* 7–29%), “articles” (*article*) (*M* = 13%, *SD* = 2%, *range* 7–20%), “indefinite pronouns” *(ipron*) (*M* = 14%, *SD* = 2%, *range* 7–21%) and “words with more than six letters” (*Sixltr)* (*M* = 18%, *SD* = 3%, *range* 12–26%).

#### VocEmoApI

The scores of the emotion dimensions *pleasantness*, *urgency* and *control* showed remarkable variability in the sample but no striking patterns (see Table 2). If the male and female study participants were considered separately, it became clear that the mean scores of the male participants differed from those of the female participants. The males (P1, P2, P4, P6, and P8) showed exclusively negative scores in the dimensions of *pleasantness* and *urgency* and exclusively positive scores for *control*. In contrast, the females showed a different grouping for the emotion dimensions: P5 showed positive scores for *pleasantness*, *urgency,* and *control*. P3 also showed positive scores for *pleasantness* and *control* and a negative score for *urgency*. The mean value (M) of the Pitch (F0) (*pitchAverage*) was 105 Hz for the male study participants and 156 Hz for the two female study participants. The value for the average pitch (F0) in general is about 120 Hz in males and about 220 Hz in females^60^.

**Table 2:** Mean distribution (M) of the emotion dimensions, prosodic features, and emotion score headline parameters for each study participant and the TC.

VocEmoApI	Study participants							
Parameters	P1	P2	P3	P4	P5	P6	P8	TC
	M (SD)	M (SD)	M (SD)	M (SD)	M (SD)	M (SD)	M (SD)	M (SD)
Dimensions								
Pleasantness	− 0.49 (0.07)	− 0.03 (0.19)	0.13 (0.10)	− 0.42 (0.10)	0.04 (0.12)	− 0.39 (0.08)	− 0.18 (0.18)	− 0.17 (0.28)
Urgency	− 0.85 (0.06)	− 0.66 (0.09)	− 0.27 (0.11)	− 0.57 (0.09)	0.60 (0.14)	− 0.58 (0.15)	− 0.68 (0.07)	− 0.43 (0.46)
Control	0.20 (0.13)	0.14 (0.12)	0.54 (0.12)	0.52 (0.11)	0.95 (0.04)	0.54 (0.19)	0.38 (0.11)	0.46 (0.28)
Prosody								
LoudnessAverage	1.10 (0.15)	1.09 (0.10)	1.20 (0.10)	1.39 (0.15)	1.86 (0.14)	1.38 (0.19)	1.28 (0.14)	1.32 (0.28)
PitchAverage	96.34 (2.97)	115.00 (6.32)	147.48 (2.34)	100.83 (3.25)	164.72 (2.98)	101.47 (4.22)	111.92 (2.98)	120.97 (24.61)
PitchVariation	7.80 (1.20)	7.80 (1.20)	6.0 (0.70)	6.40 (1.0)	11.0 (1.2)	11.0 (1.0)	6.4 (1.6)	1.74 (0.47)
SpeakingSpeed	1.58 (0.24)	1.30 (0.27)	1.43 (0.21)	1.53 (0.19)	1.49 (0.18)	1.28 (0.23)	1.36 (0.23)	1.43 (0.24)
Intensity	1.21 (0.08)	0.88 (0.13)	0.75 (0.09)	1.06 (0.05)	1.53 (0.18)	1.17 (0.09)	0.97 (0.09)	1.06 (0.26)
Category_v2_scores								
Sadness	1.91 (0.13)	1.06 (0.29)	0.24 (0.10)	1.29 (0.11)	0.01 (0.01)	1.37 (0.24)	1.23 (0.20)	0.99 (0.65)
Passion	0.02 (0.00)	0.00 (0.00)	0.26 (0.19)	0.00 (0.01)	1.98 (0.21)	0.04 (0.07)	0.00 (0.01)	0.32 (0.68)
Euphoria	0.00 (0.00)	0.00 (0.00)	0.27 (0.19)	0.00 (0.01)	2.07 (0.23)	0.05 (0.08)	0.00 (0.01)	0.34 (0.71)
Enthusiasm	0.00 (0.00)	0.00 (0.01)	0.20 (0.12)	0.00 (0.00)	1.76 (0.28)	0.03 (0.05)	0.01 (0.01)	0.28 (0.61)
Disgust	1.92 (0.19)	0.80 (0.32)	0.20 (0.08)	1.25 (0.11)	0.84 (0.19)	1.41 (0.22)	1.08 (0.18)	1.03 (0.55)
Boredom	1.59 (0.22)	1.07 (0.23)	0.52 (0.13)	0.81 (0.22)	0.08 (0.07)	1.00 (0.38)	1.16 (0.21)	0.89 (0.50)
Grief	1.62 (0.06)	1.17 (0.23)	0.31 (0.14)	1.35 (0.15)	0.01 (0.01)	1.38 (0.21)	1.36 (0.17)	1.01 (059)
Valid values (N)	17	19	19	18	16	10	16	115

*M* mean, *SD* standard deviation, *TC* total collective, *pitchAverage/pitchVariation* in Hz, *speakingSpeed* in Ss (syllables per second), *dimensions/loudnessAverage/intensity/*category_v2_scores as score values.

The speech rate (*speakingSpeed*) showed values from a minimum of 0.76 to a maximum of 2.15 syllables per second (Ss) across the entire sample. The mean (*M*) was 1.43 syllables per second (Ss) with a standard deviation (*SD*) of 0.24 Ss (see Supplementary Information under Table B.1.2.2). This result illustrates that compared to the average speaking rate of approximately four syllables per second for a healthy speaker (see Chapter 2.5.2), all participants in this study spoke more slowly on average, paused many times, and sometimes only spoke using short utterances. According to the interpretation guidelines of the developers of the VocEmoApI software, presented in Chapter 2.5.2, the characteristic values of the parameter intensity of emotions (*intensity*) for the entire sample with 115 audio recordings showed a score between a minimum of 0.53 (*emotionally coloured*) and a maximum of 1.77 (far above *highly emotional*). The average score was 1.06 with a standard deviation (*SD*) of 0.26 and was thus rated as *highly emotional* (see Supplementary Table B.1.2.1). This means that the intensity of the emotions measured in the study group was never in the range of neutral speech and that most of the study group was highly emotional throughout the study.

The examination of the mean values (*M)* for all 52 emotion categories showed that the reference range was slightly exceeded for the emotions *disgust* (*M* = 1.03, *range* 0.07–2.21, *SD* = 0.55) and *grief* (*M* = 1.01, *range* 0.00–1.73, *SD* = 0.59) (see Supplementary Information under Table B.1.2.3).

On the other hand, the descriptive representation of the individual cases in Table 2, and Table B.1.2.4 showed considerable exceedances of the reference range of the mean values.

For the male study participants, these are above all the negative emotions *disgust* (P1: *M* = 1.92, *range* 1.52–2. 21, *SD* = 0.19), *sadness* (P4: *M* = 1.29, *range* 1.04–1.43, SD = 0.11) and *grief* (P6: *M* = 1.38, *range* 1.09–1.73, *SD* = 0.21) and for the female study participant P5, the positive emotion *euphoria* (*M* = 2.07, *range* 1.58–2.38, *SD* = 0.23). Female P3 was the only participant whose value did not exceed the reference range.

### Exploratory factor analysis

#### LIWC2015

The first phase of the exploratory factor analysis consisted of a principal component analysis. Table B.2.1 (Supplementary Information) shows that the *eigenvalue rule* suggested 17 factors. These explained 74% of the total variance. The eigenvalue diagram also showed a factor number of 17 with a ‘kink’ in the eigenvalue progression of the Scree test (from the top: the number of scores assessed before the ‘kink’, see Fig. BF.1 in Supplementary Information). With a value of 0.601, the KMO value showed moderate suitability of the LIWC2015 parameters for a factor analysis (see Table B.2.2 in Supplementary Information). Only by applying the parallel analysis according to Horn^55^ could the number of factors be reduced to nine (see Table B.2.3 in Supplementary Information). Based on these findings, principal component analysis was performed a second time, with the stipulation that only nine factors be extracted. However, Table B.2.4 in the Supplementary Information shows that with nine factors, only 56% of the total variance was still explained. The KMO value was again 0.601.

The interpretation and naming of the identified factors under a ‘title term’ was facilitated by the fact that the items that formed a factor were ordered in descending order according to their factor loading. Items with the highest loading thus became more visible and were used as a hook for naming. This ranking was created in SPSS by a varimax rotation (see Table B.2.5 in Supplementary Information). Only items with loadings > 0.4 were considered for further factor formation. The items with loading below this value were removed. To check the internal consistency of the factors, a reliability test was carried out using Cronbach’s alpha. The values in Table B.2.6 (Supplementary Information) show that the measure of reliability (> 0.60) was only given for Factors F1, F2, F3, F4 and F5. The remaining factors (F6, F7, F8, and F9) were not considered further.

Each title for a factor should briefly and concisely reflect the best possible interpretation of its content. The challenge of finding a title was that the content was a product of statistics, which created a new combination of parameters whose common denominator had to be reflected in the title. To facilitate the interpretation of the content of the factors of LIWC2015 (F_liwc_), a table was created in which the parameters in the respective factors are listed according to descending loading amount (see Table B.2.7 in Supplementary Information).

The extracted factors describe the ‘attention’ in its orientation and with a reference to the topic (F1_liwc_ *Presence* and F4_liwc_ *Autobiography*), the ‘thinking style’ of the study participants (F3_liwc_ *Self-critical analysis* and F5_liwc_ *Claim & Ambition*) and the ‘social relationships’ (F2_liwc_ *Social inclusion*) from the transcribed interviews.

#### Emotions scores: category_v2_scores

All 52 parameters were used for the explorative factor analysis of the category_v2_scores. In the first phase of the explorative factor analysis, a principal component analysis was carried out. As shown in Table B.3.1 (Supplementary Information), the ‘eigenvalue rule’ suggested five factors. These five factors explained 96% of the total variance. An inspection of the eigenvalue plot also indicated the ‘kink’ in the eigenvalue plot of the Scree test at a factor count of five (see Fig. BF.2 in Supplementary Information).

The suitability of the output parameters for an explorative factor analysis could again be calculated by the KMO value. The value of 0.884 (see Table B.3.2 in Supplementary Information) showed a high suitability of the variables for factor analysis. To determine the number of factors, a parallel analysis according to Horn^55^ was also carried out. This also recommended (see Table B.3.3 in Supplementary Information) five factors, since the eigenvalues of the empirical data for the first five factor possibilities were larger than the eigenvalues of the random data. In SPSS Version 25, the items of the factors were again ranked by a varimax rotation so that the items with the highest loading for the naming process could be captured immediately (see Table B.3.4 in Supplementary Information). Only items with loadings > 0.4 were considered for the factor formation. To check the internal consistency of the factors, a reliability test was carried out using Cronbach’s alpha. The values in Table B.3.5 (Supplementary Information) show that the measure of reliability (> 0.6) was given for the Factors F1_emo_, F2_emo_, F3_emo_, F4_emo_ and F5_emo_.

To create the best titles for the factors that reflect their complex contents as much as possible in one ‘keyword’, a table was first created in which the parameters under the factor were presented in descending order (see Table B.3.6 in Supplementary Information).

Two reactive opposite action factors (F1_emo_ *Extraversion* and F2_emo_ *Introversion*), a self-reflexive frustration factor (F3_emo_ *Frustration*), a reactive negative action factor (F4_emo_ *Stress, Panic & Anxiety*), and a self-reflexive guilt factor (F5_emo_ *Confusion & Guilt*) were developed.

These two-by-five factors from the text and voice analyses, respectively, were given behavioral and emotional psychological labels and described as dependent variables (DVs) for each study participant over time and interpreted in the context of the broader individual case analyses. These factors were used as target parameters during the study and were used as surrogate markers for changes in speech and voice.

### Single case analyses

#### Study participation and adherence

The adherence of the participants to the study approach was documented by their presence/absence (see Tables B.4.1, B.4.2 in Supplementary Information) and by the author’s written observations.

The audio recording of all seven study participants (n = 7) were evaluated. Six participants completed the study at the scheduled time after 6 months; one participant was allowed to leave the facility after 4 months, and his audio documents, which were complete up to that point, were included in the evaluation of the study. In the group art therapy, a total of 118 out of 140 pictures could be designed by the seven study participants. The rate of data loss was 16%.

Study participants who created a picture in the group also generally presented it in the interviews, so the number of pictures created did not differ significantly with the number of interviews. Of 140 possible interviews, 115 took place.

Therefore, the rate of data loss for the entire study group was 18%. The rate of data loss for P2 and P3 was only 5%; the rate of data loss was 10% for P4, 15% for P1, and finally 20% each for P5 and P8. Due to the premature departure of P6, the data loss rate here was 55% and 50% respectively, including the last interview on 29 October 2016.

The interview situation with a table microphone irritated the patients with chronic schizophrenia. The repetitive questions and narrative prompts served in principle as orientation in the interview but were also perceived as a restriction in the conversation. The ‘maintenance questions’ and the ‘concrete follow-up questions’ in the interviews proved to be a suitable means to ‘bring back’ the study participants when they ‘digressed’ from the topic, including digressions due to latent psychotic symptoms.

#### Linear regression of the LIWC2015 factors

The quantitative individual case analyses showed exclusive individual courses or individual trends during the study for the LIWC2015 factors: Factor F1_liwc_ *Presence* for P1 and P3, and Factor F4_liwc_ *Autobiography* for P2 and P3. Only P3 showed statistically significant changes in the two factors (see Table 3). Her significant increase for Factor F1_liwc_ *Presence* showed that she was better able to focus her attention on the moment and on the object (picture) during the study.

**Table 3:** Regression coefficient (b) for the LIWC2015 factors F_liwc_ over the course of the study for each study participant and the total collective (TC).

Factors	Study participants							
	P1	P2	P3	P4	P5	P6 (14)	P8	TC
F1_liwc_								
*b*	0.015	− 0.004	0.024*	0.017	1.66E−05	0.081	0.005	0.018**
*p*	0.063	0.687	0.021	0.123	0.998	0.103	0.627	0.005
*R*^2^	0.212	0.010	0.275	0.142	0.000	0.334	0.017	0.359
F2_liwc_								
*b*	0.002	− 0.030*	− 0.024	− 0.017	0.001	− 0.006	− 0.010	− 0.014
*p*	0.868	0.011	0.149	0.407	0.931	0.876	0.391	0.070
*R*^2^	0.002	0.321	0.119	0.043	0.001	0.004	0.053	0.171
F3_liwc_								
*b*	0.002	0.005	0.011	0.025	− 0.004	0.034	0.017	0.009
*P*	0.849	0.630	0.444	0.128	0.792	0.272	0.083	0.052
*R*^2^	0.003	0.014	0.035	0.139	0.005	0.169	0.199	0.194
F4_liwc_								
*b*	− 0.019	0.019	0.047*	− 5.04E−05	− 0.009	0.047	0.019	0.010
*P*	0.293	0.097	0.011	0.998	0.765	0.393	0.364	0.351
*R*^2^	0.073	0.153	0.321	0.000	0.007	0.106	0.059	0.048
F5_liwc_								
*b*	− 0.001	0.016	− 0.016	0.022	0.027	− 0.051	− 0.011	0.006
*p*	0.931	0.265	0.302	0.318	0.138	0.066	0.309	0.354
*R*^*2*^	0.001	0.072	0.062	0.062	0.150	0.404	0.074	0.048

*b* regression coefficient; significance level: p ≤ 0.05*, p ≤ 0.01**, *R*^*2*^ coefficient of determination, *P6 (14)* results for 14 weeks.

In the summary of the individual case analyses, the exceptionally large variation with sometimes extreme outlier values in the Factors F2_liwc_ *Social inclusion*, F4_liwc_ *Autobiography*, and F5_liwc_ *Claim & Ambition* reflects either the tasks and topics behind the pictures or the study participants’ own guiding themes (as with P3 to Factor F4_liwc_). For Factor F2_liwc_ *Social inclusion*, this would be, for example, the task ‘My first name’, for Factor F4_liwc_ the ‘Self-portrait’ and for Factor F5_liwc_ ‘Painting with acrylic colours’.

For the sake of completeness, linear regression analyses with the LIWC2015 target parameters were also carried out over the entire study group (TC = total collective), to capture peculiarities during the study that either could not or could not sufficiently be depicted in the individual courses. In contrast to the individual case analyses, significant changes of a LIWC2015 factor during the study were observed across the entire sample, shown in Table 3, in the sense of an increase in the so-called Factor F1_liwc_ *Presence*.

#### Linear regression of the VocEmoApI factors

In the summary of the individual case analyses, the calculated data for the VocEmoApI factors presented the picture of a clear dichotomy among the study participants (see Table 4). One group, consisting of P1, P4, and P6, showed an almost linear and ‘quasi-therapeutic’ change in at least two of the three possible voice analysis target criteria over the course of the study (hereafter named ‘Group 1 with change’). The remaining participants (P2, P3, P5, and P8) formed the second group; they did not show any statistically relevant development in the results of the voice analysis during the study (hereafter referred to as ‘Group 2 without change’).

**Table 4:** Regression coefficient (b) for the factors F_emo_ of VocEmoApI over the course of the study for each study participant and the total collective (TC).

Factors	Study participants							
	P1	P2	P3	P4	P5	P6 (14)	P8	TC
F1_emo_								
b	− 0.002*	− 0.002***	0.002	− 0.003*	− 0.009	− 0.011*	0.000	− 0.002
p	0.015	0.000	0.620	0.043	0.127	0.027	0.823	0.514
R^2^	0.335	0.532	0.015	0.232	0.158	0.525	0.004	0.024
F2_emo_								
b	0.018***	− 0.004	− 0.002	0.003	− 0.002	0.056**	− 0.006	0.003
p	0.001	0.552	0.414	0.387	0.161	0.003	0.319	0.484
R^2^	0.556	0.021	0.040	0.047	0.136	0.745	0.071	0.028
F3_emo_								
b	− 0.008**	0.002	0.002	− 0.007	0.003	− 0.007	0.005	− 0.003
p	0.004	0.709	0.497	0.088	0.341	0.440	0.325	0.124
R^2^	0.429	0.008	0.028	0.171	0.065	0.087	0.069	0.126
F4_emo_								
b	− 0.003*	0.000	0.001	− 0.011*	0.001	− 0.028	0.000	− 0.004**
p	0.021	0.628	0.565	0.023	0.409	0.066	0.623	0.003
R^2^	0.308	0.014	0.020	0.282	0.049	0.402	0.018	0.393
F5_emo_								
b	0.000	0.001	0.001	− 1.98E−05	− 0.005	0.000	− 2.33E−05	0.000
p	0.456	0.335	0.797	0.942	0.161	0.948	0.918	0.852
R^2^	0.038	0.055	0.004	0.000	0.135	0.001	0.001	0.002

*b* regression coefficient; significance level: p ≤ 0.05*, p ≤ 0.01**, p ≤ 0.001***, *R*^*2*^ coefficient of determination, *P6 (14)* results for 14 weeks.

For the participants in ‘Group 1 with changes’, the extracted emotion factors developed in the same direction. The Factors F1_emo_, F3_emo_ and F4_emo_ decreased in P1, P4 and P6, and the Factor F2_emo_ increased in P1 and P6. These changes could be statistically proven. In the other group, which included P3, P5, and P8, no changes were observed in the extracted VocEmoApI factors during the study (see Table 5). This dichotomy of the sample was initially based only on the criterion with or without change in the vocal target parameters. In a further linear regression analysis of the F scores carried out separately for Group 1 and Group 2, in addition to this distinguishing feature, other group-specific characteristics were also revealed. The results of the group-separated linear regression analysis of the F_emo_-scores (Group 1: P1, P4 and P6; Group 2: P2, P3, P5 and P8) are shown in Table 5. They show the degree of change in the scores of the factors (F_emo_) for the two groups throughout the study. First, as expected, the changes that had already been mentioned as distinguishing the two groups were observed only in group 1. These significant changes concerned the Factors F1_emo_, F2_emo_, F3_emo_ and F4_emo_. There were further distinguishing features between the two groups because of the group-separated analysis. While the Factor F3_emo_ *Frustration* in Group 1 decreased continuously over the six months, this factor increased in Group 2. This trend just missed the significance level.

**Table 5:** Regression coefficient (b) for the factors F_emo_ of VocEmoApI during study for Groups 1 and 2.

Factors	Study participants	
	Group 1	Group 2
F1_emo_		
b	− 0.004***	− 0.004
p	0.000	0.426
R^2^	0.524	0.036
F2_emo_		
b	0.018**	− 0.002
p	0.004	0.593
R^2^	0.383	0.016
F3_emo_		
b	− 0.008**	0.004
p	0.006	0.056
R^2^	0.351	0.188
F4_emo_		
b	− 0.009***	0.000
p	0.001	0.610
R^2^	0.485	0.015
F5_emo_		
b	0.000	− 0.001
p	0.421	0.551
R^2^	0.036	0.020

*b* regression coefficient, significance level: p ≤ 0.01**, p ≤ 0.001***, *R*^*2*^ coefficient of determination.

If the statistical results of the linear regression analysis for the factors of the *category_v2_scores* were evaluated over the entire group (TC), a statistically significant change (decrease) over the test period was discernible only for the Factor F4_emo_ *Stress, Panic & Anxiety* (b =  − 0.004**; p = 0.003) (see Table 4). All other factors showed no statistically relevant change (all other factors p > 0.124). Therefore, the analysis across the entire group (TC) did not bring any new findings but rather showed the expected effect that the opposite developments in the two subgroups neutralized each other in the total collective (TC).

### Dual-study approach

The validity of the study results was secured by the dual-study approach in the sense of internal control through correlations between the factors of the text and voice analysis. In the individual case analyses, only individual correlations could be shown. On the other hand, correlations were also found across the entire collective, which showed a statistically secure relationship among the factors, key parameters, and quantified individual emotions of the text and voice analysis.

In summary, the results in Table 6 show that when participants analyse their own image self-critically (F3_liwc_), there is an increase in introverted emotions (F2_emo_ *Introversion*) (r = 0.561**; p < 0.01) and a decrease in self-reflective feelings of *Confusion & Guilt* (F5_emo_) (r =  − 0.425**; p < 0.01). In contrast, when study participants talked about social and autobiographical topics in the interviews (F2_liwc_ *Social Inclusion* and F4_liwc_ *Autobiography*), there was decrease in introverted emotions (F2_emo_ *Introversion*) (r =  − 0.318**; p < 0.01; r =  − 0.431**; p < 0.01, respectively).

**Table 6:** Pearson correlation matrix between the factors of F_emo_ and F_liwc_ for the total collective (TC).

		F1_emo_	F2_emo_	F3_emo_	F4_emo_	F5_emo_	F1_liwc_	F2_liwc_	F3_liwc_	F4_liwc_	F5_liwc_
F1_emo_	Pearson correlation	1	− 0.542**	− 0.221*	0.001	0.567**	0.033	− 0.245**	− 0.361**	0.152	− 0.109
	Significance (2-sided)		0	0.018	0.988	0	0.726	0.008	0	0.105	0.246
F2_emo_	Pearson correlation		1	0.509**	− 0.062	− 0.594**	− 0.122	− 0.318**	0.561**	− 0.431**	− 0.17
	Significance (2-sided)			0	0.508	0	0.193	0.001	0	0	0.069
F3_emo_	Pearson correlation			1	0.552**	− 0.526**	− 0.423**	− 0.227*	0.168	− 0.279**	0.068
	Significance (2-sided)				0	0	0	0.015	0.073	0.003	0.468
F4_emo_	Pearson correlation				1	− 0.092	− 0.331**	0.077	− 0.190*	− 0.121	0.11
	Significance (2-sided)					0.326	0	0.414	0.042	0.198	0.243
F5_emo_	Pearson correlation					1	0.337**	0.207*	− 0.425**	0.218*	− 0.087
	Significance (2-sided)						0	0.027	0	0.019	0.357
F1_liwc_	Pearson correlation						1	0.048	− 0.103	0.035	0.126
	Significance (2-sided)							0.609	0.272	0.707	0.179
F2_liwc_	Pearson correlation							1	− 0.400**	0.108	0.117
	Significance (2-sided)								0	0.253	0.213
F3_liwc_	Pearson correlation								1	0.01	− 0.318**
	Significance (2-sided)									0.919	0.001
F4_liwc_	Pearson correlation									1	0.02
	Significance (2-sided)										0.832
F5_liwc_	Pearson correlation										1
	Significance (2-sided)										
	(valid values) N	115	115	115	115	115	115	115	115	115	115

*N* the number of valid measurement points; only the statistically significant correlations between the factors of F_emo_ and F_liwc_ are shown in bold.

*The correlation is significant at the 0.05 level (2-sided).

**The correlation is significant at the 0.01 level (2-sided).

The negative correlations detected between F1_liwc_ and F4_emo_ (r =  − 0.331**; p < 0.01) support this two-dimensional insight into the dynamics of the art therapy process in a quasi-mirror image. The moderate negative correlation of the emotion factor *Stress, Panic & Anxiety* with the LIWC2015 factor *Presence* underpins the results of the linear regression analysis from Chapters 3.3.2 and 3.3.3. This can be seen from the fact that the two factors were complementary and analogous to the findings of the correlation matrix across the entire collective.

## Discussion

The starting point of this research project was the assumption that under the conditions of therapist-guided picture reflection (TGPR), there is a change in the communication behaviour of patients with chronic schizophrenia. The aim of the art therapy study was therefore to test this basic assumption with two quantitative speech and voice analysis procedures. The extent to which the art therapy study approach and the two quantitative research instruments (LIWC2015 and VocEmoApI) were suitable for this study will be discussed below.

### Study participation and adherence

Considering the nature and chronicity of schizophrenia, the rate of data loss of 16% in the group art therapy and 18% in the interviews was relatively low, i.e., without much relevance for statistical evaluation. This fulfilled the first prerequisite for the reproducibility of the study approach.

As a further condition for reproducible study results, a source of language and voice samples that made it possible to ensure comparable emotional requirements in up to 20 consecutive interviews was created.

To ensure this, therapist-guided picture reflection (TGPR) was the chosen approach for standardized interviews, in which the catalogue of questions was specifically oriented to the peculiarities of patients with chronic schizophrenia. In this way, the emotionality of the creative process of image creation could also be captured in the patients’ voices in their later reflection regarding their pictures.

The consistent use of existing art therapy elements and the reference to indications led to the development of a variant of art therapy that was suitable for use as a study approach in providing art therapy for patients with chronic schizophrenia. The trimming to study suitability, especially through the uniform repetition of art therapy procedures, could have limited the therapeutic potential of the method, so that significant processual changes would no longer be recognized by the measuring instruments used. However, when considering both the statistical study results of the individual case analyses and the overall collective, it could be determined that the measured changes were sufficient, both quantitatively and in their respective combinations, to indicate the therapeutic potential of this art therapy study approach.

A study-typical ‘downer’ arose from the artefact of the interview situation with a table microphone for digitally recording the TGPR interviews. The microphone triggered paranoid reactions matching the clinical picture for some study participants. Similar observations were apparently made by Montag et al.^16^, who had three participants leave the art therapy group due to the study setting (fear of video recordings). Therefore, for patients with schizophrenia, this form of voice recording remains an inherent problem. Although the present art therapy study had different circumstances than other studies, e.g., those by Green et al.^13^ and Crawford et al.^15^, it showed relatively high adherence to the study approach with comparatively fewer dropouts. The chosen study approach of art therapy image development and picture reflection for patients with chronic schizophrenia was therefore feasible in principle over a long period of 6 months.

### Suitability of the research instruments

Overall, the two methods of text and voice analysis proved to be suitable research instruments for use by an art therapist to treat chronic schizophrenia patients.

However, apart from the contribution to a predominantly positive balance of the study, limitations concerning the effort of data preparation and its evaluation should be noted.

#### LIWC2015

The text analysis LIWC2015 program has already been used in studies with schizophrenia patients but never in art therapy research. For a practical application of the LIWC2015 program, the German-language electronic dictionary DE-LIWC2015^28^ would therefore have to be expanded to include art science and art therapy terms. It would also be important for potential word ambiguities to be clearly recognized in relation to the context of what was said for a complete analysis of the interviews.

The enormous range of the interviews word count (from a minimum of 87 to a maximum of 1486 words) is shown in Chapter 3.1.1. A long interview with many words, filler words, and repeated words is described by Just et al.^27^ as a typical feature in schizophrenia patients with formal thought disorders^27^. The thought and language disorders of schizophrenia patients were also highlighted in the Introduction section. Accordingly, this fluctuation could also be seen as a feature of language typical of the schizophrenia in the interview situation.

The LIWC program has been continuously developed since its development by Pennebaker et al.^51^; for the first time, the current version of the LIWC2015 software was used in this clinical picture. What distinguishes the LIWC program from other text analysis software is that it is also available and validated in the German language^28,52,61^.

For the inquiry in German language, the text analysis could be combined with German version of the LIWC2015 program, namely, the DE-LIWC2015, developed by Meier et al.^28^. The strength of this study was that an average of 90% of the words from all interviews could be assigned and evaluated. This strength confirmed the improvement of the extended DE-LIWC2015 dictionary highlighted by Meier et al.^28^, with an average word coverage of 83%^28^.

Regarding the application of this text analysis procedure in this study, the preexisting scientific results from studies with schizophrenia patients were particularly noteworthy. This procedure has not yet been used as a potential control instrument for therapeutic interventions in corresponding studies but rather to characterize linguistic features of the disorder (e.g., in the studies by Hong et al.^25^, Minor et al.^21^, Bonfils et al.^26^, and Just et al.^27^).

#### VocEmoApI

In this study, VocEmoApI technology was used for the first time in study participants with chronic schizophrenia.

For data analysis, it was helpful that this emotion recognition technology not only records the known prosodic parameters (fundamental frequency, variation in fundamental frequency, the speech rate, etc.) but also automatically assigns voice signals with emotional colouring to 52 emotionally-defined parameters (category_v2_scores) and scores them according to how well they fit into the folder. The ratings are the scores, and the emotion categories have names such as *sadness*, *anger*, or *loving*.

In their descriptive part based on the lead parameters, the voice analysis results showed an opposite vocal expression of emotion between the male and female study participants.

The male study participants’ lead parameters were *sadness*, *disgust*, and *boredom*, and positive emotions were almost never detected. When assessing the results of facial expression research, schizophrenia patients who were male showed “a severe reduction in mimic affectivity”, which could be recognized and interpreted as “a consequence of the disappearance of genuine joy”^62^. Krause^62^ describes the emotional parameters *sadness*, *disgust,* and *grief* as “negative leading affects” of schizophrenia patients. Here, facial expression and voice analyses seemed to correspond completely and allowed the consistently negative emotional expression of the five male study participants to be classified as a known emotional phenotype in the diagnostic spectrum of schizophrenia.

The two women showed conspicuous and especially contrasting presentations of the leading emotional parameters compared to the male study participants, which is an interesting finding; however, it remains an essentially unexplained phenomenon in these study results.

As further findings of the descriptive statistics, the commonalities linking all study participants should be discussed. These include the high-intensity emotions, ranging between *emotionally coloured* and *highly emotional*, and prosodic features, including a low pitch (F0) and slow speech rate. In the current state of research, emotion psychology only offers meaningful approaches for a satisfactory interpretation of the prosodic features and only in part: Murray and Arnott^63^ as well as Scherer and Wallbott^64^ associate a low average fundamental frequency (F0) and a slow rate of speech with the emotions *sadness* or *dejection*. Similar observations were also made by Stassen^18^, Perlini et al.^31^, Cohen et al.^33^, Martinez-Sánchez et al.^34^, and Parola et al.^36^. These authors were able to document that the speech samples of schizophrenia patients were characterized by increased pause times, a slower speech tempo, and a lower fundamental frequency compared to healthy control subjects. These findings would fit with the results of the five male study participants, who showed the emotions *sadness*, *disgust*, *boredom,* and *grief* as the leading parameters, among others. However, there is no explanation in this model for the prosodic characteristics of the two women included in the study. Their emotional parameters did not lie in the negative but mainly in the positive emotional spectrum, e.g., *longing* for P3 and *euphoria* for P5.

In view of the present study results, VocEmoApI technology also seemed to be generally suitable as a control instrument for psychotherapeutic interventions for chronic schizophrenia patients in the broader sense. Similarly, Cohen and Elvevåg^65^, Parola et al.^36^, and Low et al.^22^ pointed to this potential of automated voice analysis. As an advantage of GeMAPs, in their guidelines for machine learning models, Low et al.^22^ highlight that the standard GeMAPS parameter set offers the advantage of the ability to compare results with those of other acoustic feature extraction methods^22^.

The suitability of this instrument for its use in art therapy studies with schizophrenia patients was also demonstrated by the fact that the VocEmoApI technology did not require laboratory conditions for the audio recordings due to the “Voice Activity Detection” (VAD).

### Evidence for procedural changes

For the individual case analyses, only individual progressions could be shown for the LIWC2015 factors. Across the entire collective, F1_liwc_ (*Presence)* showed a statistically significant change and thus the development of increased confidence in language use. The study participants increasingly succeeded in expressing themselves ‘more precisely’, i.e., they seemed to gain confidence in language use. The initial urge to speak of individual study participants possibly points to an uncertain conversational style (e.g., using more filler words) and/or a characteristic feature in schizophrenia patients with formal thought disorders^27^. Thus, the statistically significant change in the Factor F1_liwc_ (b = 0.018**; p = 0.005) shows that there were increasingly fewer filler words (*filler*) in the interview texts of the sample over the course of the study and that speech was increasingly more precise and fluent (fewer *nonflu*). Thus, the factor could be regarded as a therapeutic criterion for a training effect and as a surrogate marker.

The results of the linear regression analysis of the VocEmoApI voice analysis factors revealed a dichotomy within the sample: study participants with a high level of distress who experienced further activation of the F2_emo_ *Introversion* factor in the protected manner of the therapist-guided picture reflection (TGPR) and study participants without distress in the voice recordings and without activation.

Across the entire sample, the results of the VocEmoApI voice analysis showed a particularly high sensitivity to change for the Factor F4_emo_ *Stress, Panic & Anxiety*. It would thus have the potential to provide surrogate markers of a sustainable activation of emotions. The activation of emotions could be recognized in a differentiated way with this method of voice analysis. This distinguishes VocEmoApI voice analysis as a research tool that could also be of general importance for art therapy, especially since the activation of emotions is considered an important effective factor of this complementary form of therapy.

The original assumption that the linguistic and vocal characteristics of patients with chronic schizophrenia would improve through repeated conversations about their own picture under the conditions of a therapist-guided picture discussion could not be confirmed across the board. The results of the linear regression analysis of the LIWC2015 factors on the individual cases nevertheless prove a procedural change in the sense of the research question of the study. However, this linguistic development was not evident in all study participants but only in individual participants (e.g., P3) or in the statistics on the overall collective. This change was reflected in the Factor F1_liwc_ (*Presence*), which was interpreted as an increase in confidence in language use and, obviously, as a training effect.

Of the study participants who showed an emotional process during the study (‘Group 1 with change’) none became ‘happier’. The emotional states of *Frustration* and *Stress, Panic & Anxiety* depicted in Factors F3_emo_ and F4_emo_ decreased significantly during the study, and the anxiety and agitation levels of the study patients were also reduced, but their emotions were directed more ‘inwards’ and they became ‘sadder’ (see Factor F2_emo_ *Introversion*).

In this construction, the language and voice analysis of the study participants should also contain the information that indicates the activation of cathartic emotions. In the case of the study participants in whom such an emotional process could be demonstrated over the six months of the study, this was ultimately successful. This variant of emotional activation is again one of the recognized components in the spectrum of general effective factors of art-based therapies^10,66^. The emotion-related information hidden in speech and voice offers itself as a completely new source of target parameters in regard to demonstrating therapeutic effects of art therapy in studies.

### Dual study approach

A special hope regarding the benefit of internal control in the study design was directed towards the dual approach of text and voice analysis. However, for the individual case analyses, only intraindividual correlations between the findings of both instruments could be shown. The dual use of text and voice analysis provided interesting insights into the linkage of thoughts, language, and emotions, but beyond that, the control function regarding an affirmative or corrective potential was only fulfilled in small proportions. Over the entire collective, the linear regression analysis showed perfectly complementary courses between F4_emo_ and F1_liwc_.

## Conclusions

This paper presents an elaborate art-therapeutic study approach for patients with chronic schizophrenia with a form of therapist-guided picture reflection (TGPR), which is proposed as a research tool for clinical studies. In addition, for the first time, the two instruments (LIWC2015 and VocEmoApI) were used in an art therapy study including patients with chronic schizophrenia, and their suitability for detecting procedural changes in study patients was evaluated in a differentiated manner. This could give new and innovative impetus to efficacy research.

However, due to the lack of control of the results achieved by baseline data (Phase A), it was not possible to establish a causal relationship between the intervention (TGPR) and the procedural change^67^. In line with these arguments, the present art-therapeutic study approach offers a plausible introduction to efficacy research ‘entry’ because it was not previously possible to prove effectiveness itself but only to test a study approach or investigative instruments for which such proof could be achieved in a combination of follow-up studies.

Should the potential of VocEmoApI voice analysis be confirmed in follow-up studies, this would be of particular importance for research on the efficacy of art therapy. In further controlled individual case studies, it might be possible to verify whether there is indeed a causal relationship between a therapist-guided picture reflection (TGPR) art therapy intervention and the demonstrated changes in the emotional responses of the study patients.

With the vocal analysis method presented here (VocEmoApI technology), for the first time, researching art therapists have a tool at their disposal that allows them to play their own role in the recruitment of study patients in addition to the purely medical selection criteria. Emotional profiles could be created for art-therapeutic study patients by using an examination instrument optimized for art-therapeutic study practice (e.g., in the form of a mobile application software). This applies not only to the acquisition of profiles in the recruitment of study participants, with which to optimize the comparability of patients in a study group but also to efficacy research in the function of variables at trial times during a therapeutic process.

### Supplementary Information

Supplementary Figures.

Supplementary Tables.

## Data Availability

All data underlying the results in this study are available without restriction at 10.5281/zenodo.592964.
