# Supplementary material for: Computerized text and voice analysis of patients with chronic schizophrenia in art therapy
Source: Sci Rep. 2023 Sep 25;13:16062. doi: 10.1038/s41598-023-43069-y (PMC10520069; doi:10.1038/s41598-023-43069-y)
Supplement: Supplementary file 1 — Supplementary Figures. [file 41598_2023_43069_MOESM1_ESM.docx]

**SUPPLEMENTARY INFORMATION (Figures)**

**BF.1 Scree-test for 61 parameters of LIWC2015**


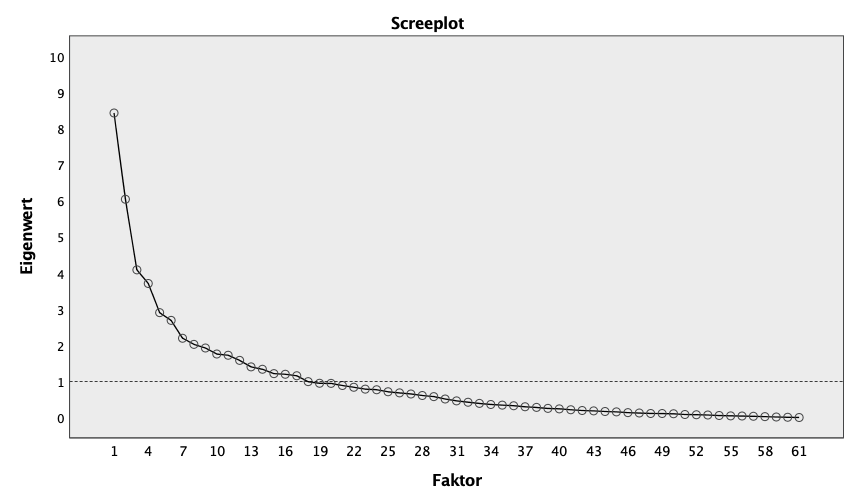


**(intrinsic value)**

**(factor)**

Figure BF.1

Scree-test for 61 parameters of LIWC2015.

**BF.2 Scree-test for 52 parameters of category_v2_scores**


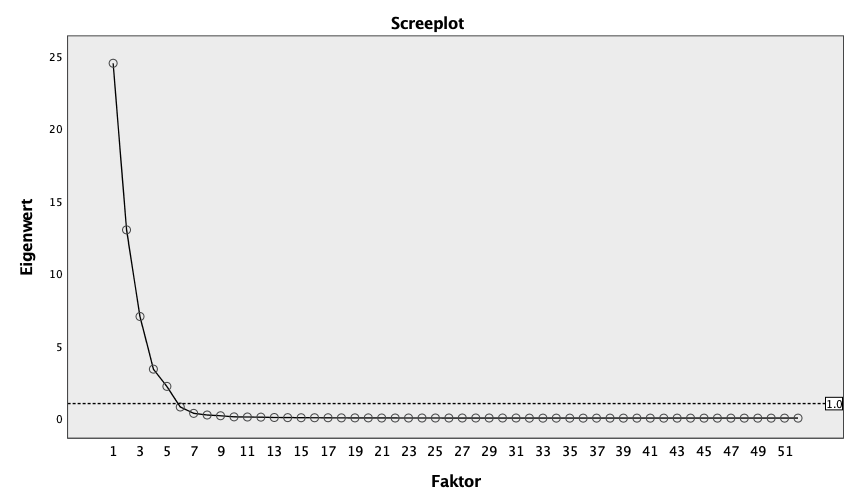


**(factor)**

**(intrinsic value)**

Figure BF.2

Scree-test for 52 parameters of category_v2_scores.
