# Supplementary material for: Computerized text and voice analysis of patients with chronic schizophrenia in art therapy
Source: Sci Rep. 2023 Sep 25;13:16062. doi: 10.1038/s41598-023-43069-y (PMC10520069; doi:10.1038/s41598-023-43069-y)
Supplement: Supplementary file 2 — Supplementary Tables. [file 41598_2023_43069_MOESM2_ESM.docx]

**SUPPLEMENTARY INFORMATION (Tables)**

**A.1 List of study participants (anonymized)**

Table A.1

*List of study participants (anonymized).*

| Patient | Birth year | Gender | Diagnosis | Move-in |
| --- | --- | --- | --- | --- |
| P1 | 1967 | M | paranoid schizophrenia (F 20.0)  mental behavioral changes due to alcohol (F10.3) | 2015 |
| P2 | 1958 | M | paranoid schizophrenia (F 20.0)  schizoaffective disorder (F 25.1) | 2015 |
| P3 | 1970 | W | paranoid-hallucinatory schizophrenia (F 20.0) | 2014 |
| P4 | 1963 | M | schizophrenic residual (F20.5) | 2002 |
| P5 | 1956 | W | paranoid-hallucinatory schizophrenia (F20.0) | 2014 |
| P6 | 1954 | M | paranoid schizophrenia (F 20.0) | 2015 |
| P8 | 1965 | M | paranoid schizophrenia (F 20.0)  schizophrenic residual (F 20.5) | 2015 |

M: male; W: female.

**A.2 Tasks and topics for creating images in the study**

Table A.2

Tasks and topics for creating images in the study.

| Date | Task/Topic | Task Description |
| --- | --- | --- |
| 1.  06.05.16 | Collage | Introduction: creating a collage  1. Select three photos on the fly.  2. Stick this on a piece of paper and design the surroundings with colored pencils of your choice. |
| 2.  13.05.16 | First-name | Introduction: first names in everyday life; What is the meaning of my first name?  1. If you like: find the meaning of your first name in the first name book and write it down on a colored button.  2. Write / design your first name on a piece of paper and design the surroundings. |
| 3.  20.05.16 | Springtime | Introduction and brainstorming: colors (1) and spring  1. Use paste to create a spring motif on a sheet of paper.  2. When it has dried, color it with felt-tip pens. |
| 4.  03.06.16 | Rainbow | Introduction and brainstorming: colors (2) and rainbows  1. Draw a rainbow on the paper with a pencil. (If you like, use a template as a guide.)  2. Color this with oil pastels as you imagine. You can then make it glow with a blue stain. |
| 5.  01.07.16 | Painting at acrylics | introduction: acrylic paints (1)  1. Choose a canvas / cardboard (different sizes available).  2. Prime these with your favorite color.  3. When the surface is dry, fix the surface with TESA-crepe and paint in the free areas. |
| 6.  8.07.16 | Hands (1) | introduction and brainstorming on the subject of 'hands'  1. Draw a pencil around both hands on a piece of paper.  2. Design the picture with color pencils. |
| 7.  15.07.16 | Hands in the group (2) | Introduction: hands on community picture  1. Select the hand that you would like to design on the group picture (`piece of cake´).  2. Cut this out, stick it on the 'piece of cake' and design the surroundings. |
| 8.  22.07.16 | My sign of the zodiac | Introduction and brainstorming on the topic of zodiac signs; What does my zodiac sign symbolize?  1. Choose a colored drawing board.  2. Design your zodiac sign as a symbol. (If you like, use a template as a guide.)  3. Prime the surfaces of the motif with paste.  4. Spread the sand on the paste and then let the picture dry. |
| 9.  29.07.16 | Collage: What gives me strength? | Introduction: What is a Resource? What things, people, etc. are good for you? What gives you strength?  1. Select three photos that symbolize strength for you.  2. Stick this on a piece of paper and design the surroundings. |
| 10.  12.08.15 | Self-portrait | Introduction: Self-portrait  paint / draw yourself. Use the colors of your choice (e.g., crayons or felt-tip pens). |
| 11.  19.08.16 | Mandala (with and without template) | Introduction: Mandala (design options)  Variant 1: Design a mandala according to your ideas.  Option 2: Choose a mandala from the templates and paint it. |
| 12.  26.08.16 | Community Picture | Introduction: community picture  1. Choose your favorite color from the oil pastels.  2. Draw a symbol for your person on the picture.  3. Then pass the picture to your partner on the left.  4. Take over the picture of your seat partner on the right and add to his or her picture.  5. Your picture is finished when it comes back to you. |
| Date | Task/Topic | Task Description |
| 14.  09.09.16 | Good-star | Introduction and brainstorming: What are good for me? What resources / strengths do I have? What am I particularly good at?  1. Draw (with assistance) a five-pointed star.  2. Draw / paint / write a symbol for what is good for you in each point. |
| 15.  16.09.16 | Painting at water colors | Introduction: Watercolors (1)  Variant 1: Guided experimentation with watercolors  Variant 2: Design freely with watercolors. |
| 16.  23.09. | Power-animal | Introduction and brainstorming: characteristics of animals  1. Select an animal photo from the collage material that symbolizes strength for you. Glue it on a piece of paper and design the surroundings. |
| 17.  30.09.16 | Tree | Introduction and brainstorming: tree  1. Draw a tree on a piece of paper in pencil. Color it if you like.  2. Use watercolors (2) to paint a tree on the sheet of paper (based on the previous week). |
| 18.  14.10.16 | Painting with acrylic paints (1): calendar pictures | Introduction: preliminary drawing / acrylic paints (2)  1. Choose a calendar sheet.  2. Draw the subject on a canvas. |
| 19.  21.10.16 | Painting with acrylic paints (2): calendar pictures | introduction: painting with acrylic paints (2)  Paint / design the calendar picture motif on the canvas with acrylic paints. |
| 20.  29.10.16 | Collage: What do I take with me? -  What am I leaving there? | Introduction and thoughts on parting  1. Select two photos: one for what you want to take with you from art therapy and one for what you want to leave.  2. Glue the photos onto a sheet of paper and design the surroundings. |
|  | Rondo | 3. Compose a rondo for your collage and present it to the group if you like.  The rondo is structured as follows:  1. line  2. line  3. line  4. line like 1st line  5. line  6. line  7. line  8. line like 1. and 4. line  9. like the 2. line |

**A.3 Structure: TGPR (*Therapist guided picture reflection)***

Table A.3

*Structure of the TGPR according to Misoch (2015).*

| 1. Information phase  First, the author greets the study participant and thanks him for taking the time. All study participants are advised that they can take their time to think about the answer. The study participants have already been informed about the author's duty of confidentiality. In addition to providing information about voluntariness, this is emphasized again.  The "information phase" takes place before the start of each TGPR and is part of the ritual.  2. Warm-up and entry phase (*Warm-up)*  This is followed by a "warm-up phase". The “warm-up and entry phase” is particularly important in this setting, consisting of several interviews over weeks and months. The study participants must motivate themselves again and again and get involved in the picture review and must not have the feeling that they are "working through" the questions. The design of the "warm-up and entry phase" is to be handled individually.  3. Main phase with eight key questions  During the “main phase”, the relevant topics are discussed in a communicative exchange with the interviewee (Misosch, 2015, p. 68). The art therapy guidelines developed from previous knowledge (see A.8) are used here.  4. Closing and closing phase  Would you like to say something else about the picture?  The interview is completed in the “closing and closing phase”. With the question "Would you like to say something else about the picture?", The study participant can contribute open thoughts and topics to the picture discussion. |
| --- |

**A.4 Final version of TGPR**

Table A.4

*Final version of TGPR.*

|  | Key question or narrative request | Content aspects | Concrete inquiries | Maintenance issues | What does the question aim at? |
| --- | --- | --- | --- | --- | --- |
| 1. | First, I would like you to look at the picture for a moment. (Break)  Please describe how the picture affects you. | Get a second (intuitive) impression or overall impression  (distant perspective) | What do you notice in particular?  What are you surprised?  What is there? And since? | non-verbal maintenance  Do you notice anything else? | intentional perception |
| 2. | Please tell us what you depicted in the picture? | Description of the visible:  Motif, theme, figures, shapes, material, color, etc. | What do you see?  What is happening now?  And here? | non-verbal maintenance  Is there anything else?  And otherwise? | individual perception |
| 3. | Please tell how the picture was created. | How was the image developed in relation to?:  Technology, color, space, shape, surface, lines, theme, composition, representational / abstract | What did you do first?  And then?  Which technology, colors, ... did you choose?  Was there a topic?  Did you use a template? | non-verbal maintenance  What happened then?  Is there anything else?  And otherwise?  And then? | self-organisation  backward facing reflection  Memory ability |
| 4. | Please describe how you felt while painting or designing. | How did the study participant experience the process?  (Focus on reflecting on the creative process) | What was easy?  What was hard?  How are you with  trouble  handled? | non-verbal maintenance  Is there anything else?  And otherwise? | phenomenological  `Development´ of feelings |

|  | Key question or narrative request | Content aspects | Concrete questions | Maintenance questions | What does the question aim at? |
| --- | --- | --- | --- | --- | --- |
| 5. | Please tell us what the meaning of the picture is for you. | How does the study participant assess his picture?  The focus is on the intent of the study participant; The study participant's own image interpretations are encouraged | What does this figure, surface, color, ... mean?  What made you move this figure, color, ...  to choose?  Can you tell us more about it?  What does this figure, color, ... mean for you? | non-verbal maintenance  Is there anything else?  What else? |  |
| 6. | If you wanted to give your picture a title, what could it be called? | Bundling (essence) of the inside and outside perspective of the study participant in the title | What do you think of spontaneously? | non-verbal maintenance  Do you have any other ideas? | Appreciation of the image and its creator  Ability to abstract through language |
| 7. | To what extent are you satisfied with the picture (result)? | Activate self-awareness; Verbalize idiosyncrasies and abilities | What do you like best?  What do you like less?  Can you tell why? | non-verbal maintenance  Is there anything else?  Do you have an example of this?  Can you explain that in more detail? | Strengthening the ego-function  Question as an indicator of successful handling with problems and obstacles |
| 8. | Please look at the picture for a moment.  (Break)  How does the picture affect you now? | Checking the second impression.  Reflection, discussion of open questions | Has anything changed?  If 'yes': what is different?  Would you do it differently today?  If 'yes': what would you do differently? | non-verbal maintenance  Is there anything else?  What else? | Review of previous perception for  AT image creation or in the course of the conversation |
|  |  |  |  |  |  |
|  |  |  |  |  |  |

# A.5 LIWC2015

Table A.5

LIWC2015 Output Variable Information [51].

| **LIWC2015-Category** | **Output** | **Examples** |
| --- | --- | --- |
| **Word Count** | **WC** |  |
| **Words/sentence** | **WPS** |  |
| **Word>6 letters** | **Sixltr** |  |
| **Dictionary words** | **Dic** |  |
|  |  |  |
| *Summary language Varilabes* |  |  |
| Analytical thinking | Analytic |  |
| Clout | Clout |  |
| Authentic | Authentic |  |
| Emotional tone | Tone |  |
|  | | |
| 1. ***Linguistic Dimensions*** | | |
| *Total function words* | *funct* | it, to, no |
| *Total pronouns* | *pronoum* | I, them |
| *Personal pronouns* | *ppron* | I, them |
| **1st pers singular** | **i** | I, me |
| **1st pers plural** | **we** | we, us |
| **2nd person** | **you** | you, thou |
| **3rd pers singular** | **shehe** | she, her |
| **3rd pers plural** | **they** | they, their |
| **Impersonal pronous** | **ipron** | it, it´s |
| **Articles** | **article** | a, an, the |
| **Prepositions** | **prep** | to, with |
| **Auxiliary verbs** | **auxverb** | am, will |
| **Common Adverbs** | **adverb** | very, really |
| **Conjunctions** | **conj** | and, but |
| **Negations** | **negate** | no, not |
| ***Other Grammar*** |  |  |
| **Common verbs** | **verb** | eat, come |
| **Common adjectives** | **adj** | free, long |
| **Comparisons** | **compare** | greater, best |
| **Interrogatives** | **interrog** | how, when |
| **Numbers** | **number** | secound |
| **Quantifiers** | **quant** | few, many |
|  | | |
| 1. ***Psychological Processes*** | | |
| *Affective processes* | *affect* |  |
| **Positive emotion** | **posemo** | happy, cried |
| **Negative emotion** | **negemo** | love, nice |
| **Anxiety** | **anx** | worried |
| **Anger** | **anger** | hate, kill |
| **Sadness** | **sad** | crying, grief |
|  |  |  |
| *Social Processes* | *social* |  |
| **Family** | **family** | dad, daughter |
| **Friends** | **friend** | buddy |
| **Female references** | **female** | girl, her |
| **Male references** | **male** | boy, his, dad |
| *Cognitive processes* | *cogproc* |  |
| **Insight** | **insight** | think, know |
| **Causation** | **cause** | because |
| **Discrepancy** | **discrep** | should |
| **Tentative** | **tentat** | maybe |
| **Certainy** | **certain** | always |
| **Differentiation** | **differ** | hasn´t, but |
| *Perceptual processes* | *percept* |  |
| **See** | **see** | view, saw |
| **Hear** | **hear** | listen |
| **Feel** | **feel** | Feels, touch |
|  |  |  |
| *Biological processes* | *bio* |  |
| **Body** | **body** | hands, cheek |
| **Health** | **health** | clinic, flu |
| **Sexual** | **sexual** | horny, love |
| **Ingestion** | **ingest** | dish, eat |
|  |  |  |
| *Drives* | *drives* |  |
| **Affiliation** | **affiliation** | ally, friend |
| **Achievement** | **achieve** | win, better |
| **Power** | **power** | bully |
| **Reward** | **reward** | take, prize |
| **Risk** | **risk** | danger |
|  |  |  |
| *Time orientations* | *TimeOrient* |  |
| **Past focus** | **focuspast** | ago, did |
| **Present focus** | **focuspresent** | today, is |
| **Future focus** | **focusfuture** | may, will |
|  |  |  |
| *Relativity* | *relative* |  |
| **Motion** | **motion** | arrive, go |
| **Space** | **space** | down, in |
| **Time** | **time** | end, until |
|  |  |  |
| Personal concerns |  |  |
| **Work** | **work** | job, majors |
| **Leisure** | **leisure** | cook, chat |
| **Home** | **home** | kitchen |
| **Money** | **money** | audit, cash |
|  |  |  |
| **Religion** | **relig** | altar, church |
| **Death** | **death** | bury, kill |
|  |  |  |
| *Informal language* | *informal* |  |
| **Swear words** | **swear** | fuck, damm |
| **Netspeak** | **netspeak** | btw, lol, thx |
| **Assent** | **assent** | agree, OK |
| **Nonfluencies** | **nonflu** | er, hm, umm |
| **Fillers** | **filler** | Imean, youknow |
|  | | |
| 1. ***Punctuation*** | | |
| Total Punctuation |  |  |
| Periods |  |  |
| Commas |  |  |
| Colons |  |  |
| Semicolons |  |  |
| Question marks |  |  |
| Exclamation marks |  |  |
| Dashes |  |  |
| Quotation marks |  |  |
| Apostrophes |  |  |
| Parentheses |  |  |
| Other punctuation |  |  |

Note: **Bold** = 61 study relevant LIWC2015 categories for exploratory factor analysis.

**A.6 VocEmoApI**

Table A.6
VocEmoApI Output Variable Information [53]

| **VocEmoApI-Output** |  |
| --- | --- |
| (1) general |  |
| (2) segments |  |
| (3) category_score | **v1_scores** |
| **(4) category_v2**  ***intensity*** | **v2_scores** |
| **(5) *cagory_v2_scores***  ***admiration***  ***agitation***  ***agony***  ***amusement***  ***anger***  ***anxiety***  ***badtemper***  ***boredom***  ***cheerfulness***  ***compassion***  ***contentment***  ***dejection***  ***delight***  ***desire***  ***despair***  ***disappointment***  ***disgust***  ***displeasure***  ***enthusiasm***  ***euphoria***  ***excitement***  ***fear***  ***frustration***  ***grief***  ***guilt***  ***happiness***  ***highstrung***  ***humiliation***  ***hurt***  ***impressed***  ***interest***  ***irritation***  ***longing***  ***loving***  ***moved***  ***nervousness***  ***outrage***  ***panic***  ***passion***  ***pleasure***  ***pride***  ***regret***  ***relief***  ***resentment***  ***sadness***  ***serenity***  ***shock***  ***stress***  ***suffering***  ***surprise***  ***worry*** | **v2-Scores** |
| **(6) *dimensions***  ***control***  ***pleasantness***  ***urgency*** |  |
| (7) states |  |
| **(8) *prosody***  ***loudnessAvarage***  ***pitchAvarge***  ***pitchVariation***  ***speakingSpeed*** |  |
| (9) *speaker* |  |
| (10) *speech* |  |
| Note: **Bold** = 52 category_v2_scores (line 5) for exploratory factor analysis; also, all other study-relevant categories: intensity (line 4), dimensions (line 6), and prosody (line 8). | |

# B ABOUT RESULTS

## B.1 Descriptive statistics (of all)

**B.1.1 LIWC2015**

**B.1.1.1 Range of values for 61 parameters**

Table B.1.1.1

*Range of values for 61 parameters + Dic for LIWC2015 (of all).*

| Descriptive statistics | | | | | |
| --- | --- | --- | --- | --- | --- |
| LIWC2015-parameters | *N* | *Min* | *Max* | *M* | *SD* |
| *WC(n)* | 115 | 87 | 1486 | 467 | 247 |
| *WPS* | 115 | 4 | 11 | 7 | 1 |
| *Sixltr* | 115 | 12 | 26 | 18 | 3 |
| *i* | 115 | 2 | 13 | 6 | 2,10 |
| *we* | 115 | 0 | 2,21 | 0,49 | 0,55 |
| *you* | 115 | 0 | 1,05 | 0,12 | 0,21 |
| *shehe* | 115 | 0,38 | 7,58 | 2,99 | 1,38 |
| *they* | 115 | 0,00 | 2,62 | 0,43 | 0,49 |
| *ipron* | 115 | 6,78 | 21,39 | 13,76 | 2,55 |
| *article* | 115 | 7,19 | 19,60 | 12,90 | 2,55 |
| *prep* | 115 | 1,15 | 10,02 | 6,19 | 1,70 |
| *auxverb* | 115 | 5,82 | 20,60 | 12,35 | 2,50 |
| *adverb* | 115 | 0,77 | 8,39 | 3,79 | 1,58 |
| *conj* | 115 | 6,78 | 29,39 | 17,57 | 3,30 |
| *negate* | 115 | 0,00 | 5,36 | 2,17 | 1,20 |
| *verb* | 115 | 10,86 | 25,09 | 17,88 | 2,99 |
| *adj* | 115 | 4,24 | 15,71 | 9,16 | 2,27 |
| *compare* | 115 | 1,79 | 9,68 | 5,00 | 1,62 |
| *interrog* | 115 | 0,00 | 5,65 | 1,58 | 0,92 |
| *number* | 115 | 0,00 | 4,03 | 1,19 | 0,72 |
| *quant* | 115 | 0,78 | 8,62 | 3,34 | 1,44 |
| *posemo* | 115 | 1,61 | 19,54 | 7,86 | 3,47 |
| *negemo* | 115 | 0,00 | 3,45 | 0,99 | 0,68 |
| *family* | 115 | 0,00 | 4,74 | 0,77 | 1,10 |
| *friend* | 115 | 0,00 | 1,44 | 0,19 | 0,31 |
| *female* | 115 | 0,00 | 2,08 | 0,37 | 0,48 |
| *male* | 115 | 0,00 | 3,06 | 0,12 | 0,37 |
| *insight* | 115 | 1,75 | 11,49 | 5,00 | 1,92 |
| *cause* | 115 | 0,82 | 7,63 | 3,90 | 1,50 |
| *discrep* | 115 | 0,51 | 8,43 | 3,93 | 1,90 |
| *tentat* | 115 | 0,19 | 18,71 | 4,71 | 3,15 |
| *certain* | 115 | 0,00 | 7,14 | 3,05 | 1,32 |
| *differ* | 115 | 0,00 | 6,28 | 3,01 | 1,38 |
| *see* | 115 | 0,46 | 13,41 | 4,23 | 2,40 |
| *hear* | 115 | 0,00 | 3,46 | 0,64 | 0,52 |
| *feel* | 115 | 0,00 | 2,55 | 0,80 | 0,54 |
| *body* | 115 | 0,00 | 9,36 | 1,33 | 1,67 |
| *health* | 115 | 0,00 | 1,90 | 0,29 | 0,43 |
| *sexual* | 115 | 0,00 | 2,75 | 0,38 | 0,55 |
| *ingest* | 115 | 0,00 | 3,18 | 0,28 | 0,53 |
| *affiliation* | 115 | 0,00 | 4,60 | 1,03 | 0,94 |
| *achiev* | 115 | 2,24 | 10,73 | 4,89 | 1,66 |
| *power* | 115 | 0,00 | 5,43 | 1,61 | 1,02 |
| *reward* | 115 | 0,00 | 5,08 | 1,26 | 1,22 |
| *risk* | 115 | 0,00 | 2,33 | 0,46 | 0,51 |
| *focuspast* | 115 | 1,02 | 9,36 | 3,76 | 1,32 |
| *focuspresent* | 115 | 6,74 | 19,54 | 12,72 | 2,60 |
| *focusfuture* | 115 | 0,00 | 5,06 | 1,31 | 0,82 |
| *motion* | 115 | 0,00 | 3,41 | 1,27 | 0,76 |
| *space* | 115 | 1,88 | 11,62 | 6,10 | 1,96 |
| *time* | 115 | 0,98 | 8,43 | 4,06 | 1,48 |
| *work* | 115 | 1,79 | 10,38 | 4,87 | 1,59 |
| *leisure* | 115 | 0,00 | 4,80 | 1,35 | 0,98 |
| *home* | 115 | 0,00 | 1,87 | 0,26 | 0,39 |
| *money* | 115 | 0,00 | 2,15 | 0,28 | 0,39 |
| *relig* | 115 | 0,00 | 2,34 | 0,37 | 0,46 |
| *death* | 115 | 0,00 | 2,00 | 0,11 | 0,27 |
| *swear* | 115 | 0,00 | 0,61 | 0,04 | 0,11 |
| *assent* | 115 | 0,75 | 9,25 | 3,56 | 1,75 |
| *nonflu* | 115 | 0,00 | 13,47 | 1,73 | 2,05 |
| *filler* | 115 | 0,00 | 16,04 | 2,64 | 2,83 |
| *Dic* | 115 | 82 | 96 | 90 | 3 |
| valid values (list values) | 115 |  |  |  |  |

Note: *N* = number of evaluations; *M* = mean; *SD* = Standard deviation; *WC* (*Word count*) = Number of words used; all other output parameters in %; *Dic* (*Dictionary words*) = percentage of recognized words.

**B.1.1.2 Mean values of LIWC2015**

Table B.1.1.2

Mean values (M) of the 61 LIWC2015 parameters of all participants.

| LIWC2015-parameters | P1 (*M*) | P2 (*M)* | P3 (*M*) | P4 (*M*) | P5 (*M*) | P6 (*M)* | P8 (*M)* |
| --- | --- | --- | --- | --- | --- | --- | --- |
| *WC* | 409,24 | 644,53 | 401,32 | 362,50 | 547,56 | 686,70 | 298,12 |
| *WPS* | 5,68 | 6,79 | 5,29 | 6,38 | 8,39 | 8,39 | 7,40 |
| *Sixltr* | **14,91** | **17,10** | **17,46** | **19,02** | **17,07** | **21,72** | **19,41** |
| *i* | 5,84 | **7,64** | **7,77** | 5,68 | 5,97 | 5,24 | 5,58 |
| *we* | 0,40 | 0,30 | 0,85 | 0,45 | 0,38 | 0,25 | 0,67 |
| *you* | 0,06 | 0,34 | 0,09 | 0,12 | 0,06 | 0,05 | 0,05 |
| *shehe* | 2,46 | 2,53 | 3,56 | 2,74 | 4,45 | 2,41 | 2,60 |
| *they* | 0,49 | 0,33 | 0,71 | 0,30 | 0,46 | 0,59 | 0,14 |
| *ipron* | **16,30** | **13,24** | **13,02** | **12,92** | **14,64** | **12,19** | **13,59** |
| *article* | **14,78** | 11,69 | 12,42 | **13,94** | **12,64** | 11,92 | 12,62 |
| *prep* | 5,22 | 6,67 | 4,63 | 6,61 | 6,21 | 8,50 | 6,60 |
| *auxverb* | **13,93** | **12,86** | **14,43** | **11,21** | **12,44** | 9,17 | **10,81** |
| *adverb* | 3,30 | 5,42 | 2,81 | 2,62 | 4,05 | 3,99 | 4,45 |
| *conj* | **20,37** | **18,62** | **15,66** | **16,02** | **20,90** | **14,05** | **16,22** |
| *negate* | 3,37 | 2,42 | 1,49 | 1,67 | 1,49 | 2,60 | 2,41 |
| *verb* | **19,08** | **19,90** | **20,09** | **16,20** | **17,98** | **15,20** | **15,03** |
| *adj* | **8,10** | **9,23** | **11,07** | **10,21** | **9,48** | 6,34 | 8,22 |
| *compare* | 4,39 | 5,89 | 3,87 | 6,24 | 5,07 | 4,44 | 4,82 |
| *interrog* | 2,09 | 1,95 | 0,93 | 1,89 | 2,04 | 1,02 | 0,90 |
| *number* | 1,09 | 1,27 | 1,06 | 1,71 | 1,09 | 0,95 | 1,04 |
| *quant* | 2,50 | 3,21 | 3,09 | 2,21 | 4,87 | 2,91 | 4,70 |
| *posemo* | **5,69** | **5,90** | **12,38** | **8,92** | **9,27** | 3,58 | **7,22** |
| *negemo* | 1,31 | 0,97 | 0,96 | 0,80 | 0,56 | 1,21 | 1,18 |
| *family* | 0,21 | 0,38 | 2,65 | 0,78 | 0,22 | 0,49 | 0,30 |
| *friend* | 0,25 | 0,32 | 0,14 | 0,10 | 0,06 | 0,14 | 0,31 |
| *female* | 0,14 | 0,23 | 0,69 | 0,60 | 0,09 | 0,54 | 0,28 |
| *male* | 0,22 | 0,05 | 0,27 | 0,09 | 0,03 | 0,15 | 0,03 |
| *insight* | 3,70 | 3,62 | 7,36 | 6,44 | 4,23 | 3,49 | 5,29 |
| *cause* | 5,04 | 3,36 | 2,92 | 3,93 | 4,95 | 2,95 | 4,01 |
| *discrep* | **5,72** | 5,96 | 2,51 | 3,50 | 3,20 | 3,74 | 2,66 |
| *tentat* | 2,90 | 4,89 | 0,91 | 5,69 | 3,87 | **11,68** | 6,30 |
| *certain* | 3,99 | 3,11 | 2,65 | 2,27 | 3,99 | 3,12 | 2,37 |
| *differ* | 3,79 | 4,30 | 2,06 | 2,21 | 2,62 | 2,67 | 3,31 |
| *see* | 3,85 | 2,67 | 6,89 | 6,22 | 3,12 | 1,62 | 3,80 |
| *hear* | 0,65 | 0,86 | 0,43 | 0,58 | 0,89 | 0,65 | 0,40 |
| *feel* | 0,74 | 0,59 | 1,07 | 1,06 | 0,98 | 0,36 | 0,61 |
| *body* | 1,29 | 1,34 | 1,67 | 1,69 | 0,58 | 1,42 | 1,25 |
| *health* | 0,35 | 0,42 | 0,41 | 0,07 | 0,06 | 0,73 | 0,14 |
| *sexual* | 0,07 | 0,19 | 0,88 | 0,60 | 0,18 | 0,29 | 0,32 |
| *ingest* | 0,38 | 0,29 | 0,33 | 0,39 | 0,15 | 0,27 | 0,11 |
| *affiliation* | 0,68 | 0,96 | 1,91 | 1,10 | 0,71 | 0,58 | 0,93 |
| *achiev* | 4,56 | 4,30 | 4,35 | 6,89 | 4,47 | 3,44 | 5,66 |
| *power* | 1,22 | 0,87 | 2,75 | 1,29 | 1,82 | 1,84 | 1,55 |
| *reward* | 0,76 | 0,48 | 3,11 | 0,86 | 2,14 | 0,40 | 0,67 |
| *risk* | 0,38 | 0,43 | 0,56 | 0,66 | 0,22 | 0,34 | 0,54 |
| *focuspast* | 2,88 | 4,07 | 4,22 | 3,35 | 4,59 | 3,00 | 3,85 |
| *focuspresent* | **14,76** | **13,79** | **14,88** | **10,92** | **12,39** | **10,58** | **10,44** |
| *focusfuture* | 1,54 | 1,63 | 0,97 | 1,43 | 1,07 | 1,80 | 0,91 |
| *motion* | 1,47 | 1,69 | 1,14 | 1,45 | 1,04 | 1,16 | 0,84 |
| *space* | 5,63 | **7,28** | 4,57 | 5,16 | 5,66 | 8,40 | 7,09 |
| *time* | 4,62 | 4,56 | 2,65 | 3,91 | 4,34 | 4,68 | 4,07 |
| *work* | 4,18 | 4,73 | 4,01 | 6,75 | 4,41 | 4,23 | 5,50 |
| *leisure* | 1,93 | 1,65 | 0,86 | 1,15 | 1,31 | 1,06 | 1,38 |
| *home* | 0,21 | 0,55 | 0,26 | 0,20 | 0,05 | 0,25 | 0,25 |
| *money* | 0,38 | 0,51 | 0,22 | 0,20 | 0,35 | 0,22 | 0,02 |
| *relig* | 0,29 | 0,42 | 0,13 | 0,57 | 0,32 | 0,72 | 0,29 |
| *death* | 0,26 | 0,11 | 0,03 | 0,18 | 0,01 | 0,22 | 0,00 |
| *swear* | 0,02 | 0,04 | 0,10 | 0,02 | 0,00 | 0,13 | 0,00 |
| *assent* | 5,11 | 3,52 | 2,65 | 2,76 | 1,99 | 4,47 | 4,92 |
| *nonflu* | 0,26 | 0,46 | 0,37 | 2,08 | 1,71 | 6,46 | 3,08 |
| *filler* | 0,98 | 0,82 | 0,78 | 2,74 | 1,96 | **9,64** | 4,98 |
| Note: *M* = mean; *WC* (*nominal* w*ord count)*; all other output parameters in %; key parameters = bold. | | | | | | | |

**B.1.2 VocEmoApI**

**B.1.2.1 VocEmoApI: Emotion intensity (of all)**

Table B.1.2.1

Intensity of emotions VocEmoApI, range of values.

| Descriptive statistics | | | | | |
| --- | --- | --- | --- | --- | --- |
| Intensity of emotions | *N* | *Min* | *Max* | *M* | *SD* |
| *intensity* | 115 | 0.53 | 1.77 | 1.06 | 0.26 |
| valid values (list values) | 115 |  |  |  |  |

Note: N = number of evaluations; M = mean; SD = standard deviation.

**B1.2.2 VocEmoApI: Prosodic (of all)**

Table B.1.2.2

| Descriptive statistics | | | | | |
| --- | --- | --- | --- | --- | --- |
| Prosodic | *N* | *Min* | *Max* | *M* | *SD* |
| *loudnessAverage* | 115 | 0,85 | 2,07 | 1,32 | 0,28 |
| *pitchAverage* | 115 | 20 | 36,85 | 26,34 | 5,37 |
| *pitchVariation* | 115 | 0,96 | 2,70 | 1,74 | 0,47 |
| *speakingSpeed* | 115 | 0,76 | 2,15 | 1,43 | 0,24 |
| Valid values (list values) | 115 |  |  |  |  |

Note: N = number of evaluations; M = mean; SD = standard deviation; Pitch is expressed in semitones relative to a base note (A0) of 27.5 Hz. The lowest value for pitch is 12 semitones (55 Hz) and the highest value is 62 semitones (~ 1000 Hz).

**B.1.2.3 VocEmoApI: category_v2_scores (of all)**

Table B.1.2.3

52 parameters of category_v2_scores, range of values.

| descriptive statistics | | | | | |
| --- | --- | --- | --- | --- | --- |
| *category_v2_scores* | *N* | *Min* | *Max* | *M* | *SD* |
| *admiration* | 115 | 0,03 | 1,17 | 0,37 | 0,21 |
| *agitation* | 115 | 0,00 | 0,72 | 0,09 | 0,15 |
| *agony* | 115 | 0,00 | 0,81 | 0,11 | 0,14 |
| *amusement* | 115 | 0,00 | 1,45 | 0,22 | 0,41 |
| *anger* | 115 | 0,00 | 2,06 | 0,45 | 0,48 |
| *anxiety* | 115 | 0,00 | 0,63 | 0,08 | 0,11 |
| *badtemper* | 115 | 0,00 | 2,08 | 0,42 | 0,48 |
| *boredom* | 115 | 0,01 | 1,90 | 0,89 | 0,50 |
| *cheerfulness* | 115 | 0,00 | 1,81 | 0,27 | 0,55 |
| *compassion* | 115 | 0,32 | 1,79 | 0,92 | 0,37 |
| *confusion* | 115 | 0,00 | 0,67 | 0,06 | 0,11 |
| *contentment* | 115 | 0,11 | 1,41 | 0,63 | 0,32 |
| *dejection* | 115 | 0,00 | 2,05 | 0,84 | 0,63 |
| *delight* | 115 | 0,00 | 1,10 | 0,17 | 0,29 |
| *desire* | 115 | 0,00 | 1,61 | 0,24 | 0,49 |
| *despair* | 115 | 0,00 | 1,04 | 0,26 | 0,25 |
| *disappointment* | 115 | 0,04 | 2,15 | 0,87 | 0,61 |
| *disgust* | 115 | 0,07 | 2,21 | 1,03 | 0,55 |
| *displeasure* | 115 | 0,00 | 0,97 | 0,21 | 0,21 |
| *enthusiasm* | 115 | 0,00 | 2,17 | 0,28 | 0,61 |
| *euphoria* | 115 | 0,00 | 2,38 | 0,34 | 0,71 |
| *excitement* | 115 | 0,00 | 0,92 | 0,13 | 0,25 |
| *fear* | 115 | 0,00 | 0,31 | 0,03 | 0,06 |
| *frustration* | 115 | 0,00 | 1,26 | 0,36 | 0,28 |
| *grief* | 115 | 0,00 | 1,73 | 1,01 | 0,59 |
| *guilt* | 115 | 0,00 | 0,66 | 0,06 | 0,11 |
| *happiness* | 115 | 0,00 | 1,10 | 0,17 | 0,29 |
| *highstrung* | 115 | 0,00 | 0,69 | 0,07 | 0,13 |
| *humiliation* | 115 | 0,00 | 1,00 | 0,23 | 0,20 |
| *hurt* | 115 | 0,02 | 1,63 | 0,68 | 0,49 |
| *impressed* | 115 | 0,00 | 1,08 | 0,26 | 0,17 |
| *interest* | 115 | 0,00 | 1,35 | 0,24 | 0,43 |
| *irritation* | 115 | 0,02 | 1,85 | 0,82 | 0,47 |
| *longing* | 115 | 0,03 | 1,07 | 0,45 | 0,23 |
| *loving* | 115 | 0,00 | 0,55 | 0,07 | 0,12 |
| *moved* | 115 | 0,01 | 1,84 | 0,85 | 0,46 |
| *nervousness* | 115 | 0,00 | 0,63 | 0,06 | 0,11 |
| *outrage* | 115 | 0,00 | 2,08 | 0,43 | 0,48 |
| *panic* | 115 | 0,00 | 0,62 | 0,05 | 0,08 |
| *passion* | 115 | 0,00 | 2,28 | 0,32 | 0,68 |
| *pleasure* | 115 | 0,02 | 0,90 | 0,23 | 0,17 |
| *pride* | 115 | 0,00 | 1,56 | 0,30 | 0,47 |
| *regret* | 115 | 0,00 | 0,91 | 0,38 | 0,22 |
| *relief* | 115 | 0,02 | 1,41 | 0,54 | 0,33 |
| *resentment* | 115 | 0,05 | 2,15 | 0,98 | 0,54 |
| *sadness* | 115 | 0,00 | 2,11 | 0,99 | 0,65 |
| *serenity* | 115 | 0,02 | 1,41 | 0,55 | 0,33 |
| *shock* | 115 | 0,00 | 0,81 | 0,07 | 0,12 |
| *stress* | 115 | 0,00 | 0,67 | 0,07 | 0,11 |
| *suffering* | 115 | 0,00 | 1,65 | 0,82 | 0,56 |
| *surprise* | 115 | 0,05 | 0,85 | 0,35 | 0,20 |
| *worry* | 115 | 0,00 | 0,70 | 0,06 | 0,11 |
| valid values (list values) | 115 |  |  |  |  |

Note: N = number of evaluations; M = mean; SD = standard deviation.

**B.1.2.4 VocEmoApI: mean category_v2_scores**

Table B.1.2.4

*Mean category_v2-scores of all participants.*

| ***category_v2_scores*** | P1 (*M*) | P2 (*M*) | P3 (*M*) | P4 (*M*) | P5 (M) | P6 (*M*) | P8 (*M*) |
| --- | --- | --- | --- | --- | --- | --- | --- |
| *admiration* | 0,35 | 0,44 | **0,36** | 0,15 | 0,65 | 0,26 | 0,35 |
| *agitation* | 0,02 | 0 | 0,09 | 0,1 | 0,35 | 0,12 | 0 |
| *agony* | 0,1 | 0,02 | 0,06 | 0,29 | 0,09 | 0,2 | 0,02 |
| *amusement* | 0 | 0,05 | 0,26 | 0,01 | **1,19** | 0,02 | 0,03 |
| *anger* | 0,34 | 0,08 | 0,11 | 0,61 | **1,42** | 0,56 | 0,18 |
| *anxiety* | 0,05 | 0,01 | 0,06 | 0,2 | 0,08 | 0,15 | 0,01 |
| *badtemper* | 0,3 | 0,07 | 0,11 | 0,55 | **1,43** | 0,51 | 0,15 |
| *boredom* | **1,59** | **1,07** | **0,52** | 0,85 | 0,08 | **1** | **1,16** |
| *cheerfulness* | 0 | 0 | 0,25 | 0 | **1,59** | 0,03 | 0 |
| *compassion* | **1,46** | **1,02** | **0,59** | 0,77 | 0,51 | 0,97 | **1,14** |
| *confusion* | 0,01 | 0,02 | 0,21 | 0,01 | 0,14 | 0,01 | 0,01 |
| *contentment* | 0,92 | 0,62 | **0,36** | 0,34 | **1,06** | 0,54 | 0,62 |
| *dejection* | **1,8** | 0,72 | 0,11 | **1,19** | 0,03 | **1,28** | 0,96 |
| *delight* | 0 | 0,05 | 0,23 | 0,01 | 0,85 | 0,01 | 0,03 |
| *desire* | 0 | 0 | 0,25 | 0,01 | **1,41** | 0,02 | 0 |
| *despair* | 0,29 | 0,14 | 0,07 | 0,66 | 0,04 | 0,51 | 0,19 |
| *disappointment* | **1,84** | 0,71 | 0,15 | **1,17** | 0,18 | **1,3** | 0,97 |
| *disgust* | **1,92** | 0,8 | 0,2 | **1,26** | 0,84 | **1,41** | **1,08** |
| *displeasure* | 0,29 | 0,07 | 0,06 | 0,53 | 0,06 | 0,41 | 0,12 |
| *enthusiasm* | 0 | 0 | 0,2 | 0 | **1,76** | 0,03 | 0,01 |
| *euphoria* | 0 | 0 | 0,27 | 0 | **2,07** | 0,05 | 0 |
| *excitement* | 0 | 0 | 0,17 | 0 | 0,7 | 0 | 0,01 |
| *fear* | 0,01 | 0,01 | 0,09 | 0,01 | 0,09 | 0,01 | 0 |
| *frustration* | 0,5 | 0,14 | 0,08 | 0,74 | 0,24 | 0,64 | 0,28 |
| *grief* | **1,62** | **1,17** | 0,31 | **1,36** | 0,01 | **1,38** | **1,36** |
| *guilt* | 0,01 | 0,02 | 0,21 | 0,01 | 0,15 | 0,01 | 0,01 |
| *happiness* | 0 | 0,05 | 0,23 | 0 | 0,85 | 0,01 | 0,03 |
| *highstrung* | 0 | 0 | 0,21 | 0 | 0,23 | 0 | 0 |
| *humiliation* | 0,26 | 0,17 | 0,09 | 0,51 | 0,02 | 0,42 | 0,18 |
| *hurt* | **1,25** | 0,46 | 0,1 | **1,15** | 0,15 | **1,16** | 0,75 |
| *impressed* | 0,27 | 0,4 | 0,33 | 0,12 | 0,2 | 0,18 | 0,28 |
| *interest* | 0 | 0,01 | 0,36 | 0 | **1,22** | 0,04 | 0,02 |
| *irritation* | **1,17** | 0,4 | 0,13 | **1,13** | **1,26** | **1,18** | 0,72 |
| *longing* | 0,45 | 0,66 | **0,63** | 0,29 | 0,12 | 0,34 | 0,54 |
| *loving* | 0 | 0,01 | 0,18 | 0 | 0,28 | 0 | 0,01 |
| *moved* | **1,51** | **1,02** | **0,53** | 0,75 | 0,13 | 0,92 | **1,09** |
| *nervousness* | 0,01 | 0,01 | 0,19 | 0 | 0,18 | 0,01 | 0 |
| *outrage* | 0,3 | 0,07 | 0,12 | 0,55 | **1,43** | 0,51 | 0,15 |
| *panic* | 0,02 | 0,01 | 0,06 | 0,09 | 0,06 | 0,09 | 0 |
| *passion* | 0 | 0 | 0,26 | 0 | **1,98** | 0,04 | 0 |
| *pleasure* | 0,15 | 0,33 | 0,35 | 0,08 | 0,31 | 0,13 | 0,21 |
| *pride* | 0 | 0,14 | 0,41 | 0,02 | **1,38** | 0,06 | 0,07 |
| *regret* | 0,49 | 0,38 | 0,16 | 0,64 | 0,02 | 0,58 | 0,44 |
| *relief* | 1,03 | 0,68 | 0,34 | 0,35 | 0,21 | 0,55 | 0,65 |
| *resentment* | **1,82** | 0,69 | 0,16 | **1,13** | **1,05** | **1,29** | 0,95 |
| *sadness* | **1,91** | **1,06** | 0,24 | **1,3** | 0,01 | **1,37** | **1,23** |
| *serenity* | 1,03 | 0,67 | 0,34 | 0,34 | 0,26 | 0,56 | 0,65 |
| *shock* | 0,04 | 0,01 | 0,08 | 0,19 | 0,05 | 0,16 | 0,01 |
| *stress* | 0,03 | 0,01 | 0,06 | 0,16 | 0,08 | 0,13 | 0,01 |
| *suffering* | **1,33** | 0,69 | 0,14 | **1,41** | 0,03 | **1,37** | **1,02** |
| *surprise* | 0,25 | 0,45 | **0,61** | 0,18 | 0,32 | 0,2 | 0,35 |
| *worry* | 0,02 | 0 | 0,07 | 0,12 | 0,14 | 0,12 | 0 |

Note: M = mean; key parameters = **bold**.

**B.2 Exploratory factor analysis of LIWC2015**

**B.2.1 Principal component analysis (intrinsic value**)

Table B.2.1
Principal component analysis for 61 parameters of LIWC2015 according to eigenvalue criterion.

| Explained total variance | | | | | | | | | |
| --- | --- | --- | --- | --- | --- | --- | --- | --- | --- |
| Component | initial intrinsic value | | | sums of squared  factor loadings for extraction | | | rotated sum of the  squared charges | | |
|  | Total | % of variance | cumulated % | total | % of variance | cumulated % | total | % of variance | cumulated  % |
| 1 | 8,425 | 13,811 | 13,811 | 8,425 | 13,811 | 13,811 | 7,066 | 11,583 | 11,583 |
| 2 | 6,040 | 9,902 | 23,713 | 6,040 | 9,902 | 23,713 | 4,072 | 6,675 | 18,258 |
| 3 | 4,090 | 6,704 | 30,417 | 4,090 | 6,704 | 30,417 | 3,726 | 6,108 | 24,366 |
| 4 | 3,712 | 6,086 | 36,502 | 3,712 | 6,086 | 36,502 | 3,478 | 5,701 | 30,067 |
| 5 | 2,905 | 4,762 | 41,265 | 2,905 | 4,762 | 41,265 | 3,140 | 5,148 | 35,215 |
| 6 | 2,691 | 4,412 | 45,677 | 2,691 | 4,412 | 45,677 | 2,386 | 3,912 | 39,127 |
| 7 | 2,199 | 3,606 | 49,282 | 2,199 | 3,606 | 49,282 | 2,313 | 3,791 | 42,918 |
| 8 | 2,031 | 3,329 | 52,611 | 2,031 | 3,329 | 52,611 | 2,255 | 3,697 | 46,615 |
| 9 | 1,930 | 3,164 | 55,775 | 1,930 | 3,164 | 55,775 | 2,174 | 3,565 | 50,179 |
| 10 | 1,762 | 2,889 | 58,665 | 1,762 | 2,889 | 58,665 | 2,034 | 3,334 | 53,514 |
| 11 | 1,729 | 2,834 | 61,499 | 1,729 | 2,834 | 61,499 | 1,935 | 3,173 | 56,686 |
| 12 | 1,587 | 2,601 | 64,100 | 1,587 | 2,601 | 64,100 | 1,929 | 3,163 | 59,849 |
| 13 | 1,407 | 2,307 | 66,407 | 1,407 | 2,307 | 66,407 | 1,872 | 3,069 | 62,918 |
| 14 | 1,340 | 2,197 | 68,604 | 1,340 | 2,197 | 68,604 | 1,843 | 3,022 | 65,940 |
| 15 | 1,221 | 2,002 | 70,606 | 1,221 | 2,002 | 70,606 | 1,757 | 2,881 | 68,821 |
| 16 | 1,203 | 1,973 | 72,579 | 1,203 | 1,973 | 72,579 | 1,728 | 2,832 | 71,653 |
| 17 | 1,163 | 1,906 | 74,484 | 1,163 | 1,906 | 74,484 | 1,727 | 2,831 | 74,484 |
| 18 | ,999 | 1,637 | 76,122 |  |  |  |  |  |  |
| 19 | ,954 | 1,564 | 77,686 |  |  |  |  |  |  |
| 20 | ,951 | 1,559 | 79,244 |  |  |  |  |  |  |
| 21 | ,892 | 1,462 | 80,707 |  |  |  |  |  |  |
| 22 | ,844 | 1,384 | 82,091 |  |  |  |  |  |  |
| 23 | ,792 | 1,298 | 83,389 |  |  |  |  |  |  |
| 24 | ,776 | 1,272 | 84,661 |  |  |  |  |  |  |
| 25 | ,719 | 1,179 | 85,840 |  |  |  |  |  |  |
| 26 | ,688 | 1,128 | 86,968 |  |  |  |  |  |  |
| 27 | ,659 | 1,080 | 88,048 |  |  |  |  |  |  |
| 28 | ,617 | 1,012 | 89,060 |  |  |  |  |  |  |
| 29 | ,587 | ,962 | 90,022 |  |  |  |  |  |  |
| 30 | ,519 | ,851 | 90,873 |  |  |  |  |  |  |
| 31 | ,468 | ,767 | 91,640 |  |  |  |  |  |  |
| 32 | ,431 | ,707 | 92,347 |  |  |  |  |  |  |
| 33 | ,397 | ,651 | 92,998 |  |  |  |  |  |  |
| 34 | ,369 | ,605 | 93,603 |  |  |  |  |  |  |
| 35 | ,350 | ,575 | 94,178 |  |  |  |  |  |  |
| 36 | ,335 | ,549 | 94,727 |  |  |  |  |  |  |
| 37 | ,305 | ,499 | 95,226 |  |  |  |  |  |  |
| 38 | ,287 | ,470 | 95,696 |  |  |  |  |  |  |
| 39 | ,260 | ,426 | 96,122 |  |  |  |  |  |  |
| 40 | ,248 | ,406 | 96,528 |  |  |  |  |  |  |
| 41 | ,223 | ,366 | 96,894 |  |  |  |  |  |  |
| 42 | ,202 | ,332 | 97,225 |  |  |  |  |  |  |
| 43 | ,192 | ,314 | 97,539 |  |  |  |  |  |  |
| 44 | ,174 | ,285 | 97,825 |  |  |  |  |  |  |
| 45 | ,163 | ,268 | 98,093 |  |  |  |  |  |  |
| 46 | ,143 | ,235 | 98,327 |  |  |  |  |  |  |
| 47 | ,131 | ,215 | 98,543 |  |  |  |  |  |  |
| 48 | ,120 | ,197 | 98,740 |  |  |  |  |  |  |
| 49 | ,117 | ,193 | 98,933 |  |  |  |  |  |  |
| 50 | ,111 | ,182 | 99,115 |  |  |  |  |  |  |
| 51 | ,091 | ,150 | 99,265 |  |  |  |  |  |  |
| 52 | ,085 | ,139 | 99,404 |  |  |  |  |  |  |
| 53 | ,078 | ,128 | 99,532 |  |  |  |  |  |  |
| 54 | ,063 | ,103 | 99,635 |  |  |  |  |  |  |
| 55 | ,054 | ,089 | 99,723 |  |  |  |  |  |  |
| 56 | ,049 | ,080 | 99,804 |  |  |  |  |  |  |
| 57 | ,041 | ,068 | 99,871 |  |  |  |  |  |  |
| 58 | ,032 | ,053 | 99,924 |  |  |  |  |  |  |
| 59 | ,022 | ,036 | 99,961 |  |  |  |  |  |  |
| 60 | ,014 | ,023 | 99,984 |  |  |  |  |  |  |
| 61 | ,010 | ,016 | 100,000 |  |  |  |  |  |  |
| Extraction method: principal component analysis | | | | | | | | | |

**B.2.2 KMO-test (intrinsic value)**

Table B.2.2
KMO-test as a standard test method for the suitability of the 61 output parameters of LIWC2015 for exploratory factor analysis.

| KMO-test | |
| --- | --- |
| Measure of sampling suitability according to Kaiser-Meyer-Olkin | 0,601 |

**B.2.3 Parallel analysis**

**Table B.2.3**
Parallel analysis according to Horn (1965) for 61 parameters of LIWC2015

| **Intrinsic value parallel analysis** | | |
| --- | --- | --- |
| **Intrinsic value**  **(random numbers)** | | **Intrinsic value**  **(empirical data)** |
| 1 | 2,981756 | 8,425 |
| 2 | 2,623524 | 6,040 |
| 3 | 2,484293 | 4,090 |
| 4 | 2,363282 | 3,712 |
| 5 | 2,263688 | 2,905 |
| 6 | 2,172682 | 2,691 |
| 7 | 2,085119 | 2,199 |
| 8 | 2,010733 | 2,031 |
| 9 | 1,929986 | 1,930 |
| 10 | 1,852442 | 1,762 |
| 11 | 1,785102 | 1,729 |
| 12 | 1,722223 | 1,587 |
| 13 | 1,65819 | 1,407 |
| 14 | 1,600274 | 1,340 |
| 15 | 1,543617 | 1,221 |
| 16 | 1,482126 | 1,203 |
| 17 | 1,431676 | 1,163 |
| 18 | 1,37915 | ,999 |
| 19 | 1,331504 | ,954 |
| 20 | 1,283247 | ,951 |
| 21 | 1,234256 | ,892 |
| 22 | 1,185583 | ,844 |
| 23 | 1,141639 | ,792 |
| 24 | 1,097971 | ,776 |
| 25 | 1,05634 | ,719 |
| 26 | 1,015829 | ,688 |
| 27 | 0,975683 | ,659 |
| 28 | 0,935094 | ,617 |
| 29 | 0,898453 | ,587 |
| 30 | 0,861755 | ,519 |
| 31 | 0,823775 | ,468 |
| 32 | 0,78866 | ,431 |
| 33 | 0,754719 | ,397 |
| 34 | 0,72235 | ,369 |
| 35 | 0,688335 | ,350 |
| 36 | 0,658755 | ,335 |
| 37 | 0,630883 | ,305 |
| 38 | 0,599695 | ,287 |
| 39 | 0,572549 | ,260 |
| 40 | 0,54531 | ,248 |
| 41 | 0,51403 | ,223 |
| 42 | 0,487551 | ,202 |
| 43 | 0,460596 | ,192 |
| 44 | 0,434451 | ,174 |
| 45 | 0,412027 | ,163 |
| 46 | 0,388126 | ,143 |
| 47 | 0,365397 | ,131 |
| 48 | 0,341586 | ,120 |
| 49 | 0,320015 | ,117 |
| 50 | 0,298176 | ,111 |
| 51 | 0,277565 | ,091 |
| 52 | 0,256242 | ,085 |
| 53 | 0,235948 | ,078 |
| 54 | 0,216974 | ,063 |
| 55 | 0,199367 | ,054 |
| 56 | 0,178876 | ,049 |
| 57 | 0,161178 | ,041 |
| 58 | 0,144226 | ,032 |
| 59 | 0,125183 | ,022 |
| 60 | 0,10691 | ,014 |
| 61 | 0,088205 | ,010 |

### B.2.4 Principal component analysis (9 factors)

**Table B.2.4**
Principal component analysis for 61 parameters of LIWC2015 after fixing to nine factors

| **explained total variance** | | | | | | | | | |
| --- | --- | --- | --- | --- | --- | --- | --- | --- | --- |
|  | initial intrinsic value | | | sums of squared  factor loadings for extraction | | | rotated sum of the  squared charges | | |
| component | total | % of  variance | cumulated  % | total | % of  variance | cumulated  % | total | % of  variance | cumulated  % |
| 1 | 8,43 | 13,81 | 13,81 | 8,43 | 13,81 | 13,81 | 6,96 | 11,42 | 11,42 |
| 2 | 6,04 | 9,90 | 23,71 | 6,04 | 9,90 | 23,71 | 4,64 | 7,61 | 19,02 |
| 3 | 4,09 | 6,70 | 30,42 | 4,09 | 6,70 | 30,42 | 4,53 | 7,43 | 26,45 |
| 4 | 3,71 | 6,09 | 36,50 | 3,71 | 6,09 | 36,50 | 3,66 | 6,00 | 32,45 |
| 5 | 2,91 | 4,76 | 41,27 | 2,91 | 4,76 | 41,27 | 3,64 | 5,96 | 38,41 |
| 6 | 2,69 | 4,41 | 45,68 | 2,69 | 4,41 | 45,68 | 3,18 | 5,21 | 43,62 |
| 7 | 2,20 | 3,61 | 49,28 | 2,20 | 3,61 | 49,28 | 2,60 | 4,26 | 47,88 |
| 8 | 2,03 | 3,33 | 52,61 | 2,03 | 3,33 | 52,61 | 2,45 | 4,02 | 51,90 |
| 9 | 1,93 | 3,16 | 55,78 | 1,93 | 3,16 | 55,78 | 2,36 | 3,88 | **55,78** |
| 10 | 1,76 | 2,89 | 58,67 |  |  |  |  |  |  |
| 11 | 1,73 | 2,83 | 61,50 |  |  |  |  |  |  |
| 12 | 1,59 | 2,60 | 64,10 |  |  |  |  |  |  |
| 13 | 1,41 | 2,31 | 66,41 |  |  |  |  |  |  |
| 14 | 1,34 | 2,20 | 68,60 |  |  |  |  |  |  |
| 15 | 1,22 | 2,00 | 70,61 |  |  |  |  |  |  |
| 16 | 1,20 | 1,97 | 72,58 |  |  |  |  |  |  |
| 17 | 1,16 | 1,91 | 74,48 |  |  |  |  |  |  |
| 18 | 1,00 | 1,64 | 76,12 |  |  |  |  |  |  |
| 19 | 0,95 | 1,56 | 77,69 |  |  |  |  |  |  |
| 20 | 0,95 | 1,56 | 79,24 |  |  |  |  |  |  |
| 21 | 0,89 | 1,46 | 80,71 |  |  |  |  |  |  |
| 22 | 0,84 | 1,38 | 82,09 |  |  |  |  |  |  |
| 23 | 0,79 | 1,30 | 83,39 |  |  |  |  |  |  |
| 24 | 0,78 | 1,27 | 84,66 |  |  |  |  |  |  |
| 25 | 0,72 | 1,18 | 85,84 |  |  |  |  |  |  |
| 26 | 0,69 | 1,13 | 86,97 |  |  |  |  |  |  |
| 27 | 0,66 | 1,08 | 88,05 |  |  |  |  |  |  |
| 28 | 0,62 | 1,01 | 89,06 |  |  |  |  |  |  |
| 29 | 0,59 | 0,96 | 90,02 |  |  |  |  |  |  |
| 30 | 0,52 | 0,85 | 90,87 |  |  |  |  |  |  |
| 31 | 0,47 | 0,77 | 91,64 |  |  |  |  |  |  |
| 32 | 0,43 | 0,71 | 92,35 |  |  |  |  |  |  |
| 33 | 0,40 | 0,65 | 93,00 |  |  |  |  |  |  |
| 34 | 0,37 | 0,61 | 93,60 |  |  |  |  |  |  |
| 35 | 0,35 | 0,58 | 94,18 |  |  |  |  |  |  |
| 36 | 0,34 | 0,55 | 94,73 |  |  |  |  |  |  |
| 37 | 0,31 | 0,50 | 95,23 |  |  |  |  |  |  |
| 38 | 0,29 | 0,47 | 95,70 |  |  |  |  |  |  |
| 39 | 0,26 | 0,43 | 96,12 |  |  |  |  |  |  |
| 40 | 0,25 | 0,41 | 96,53 |  |  |  |  |  |  |
| 41 | 0,22 | 0,37 | 96,89 |  |  |  |  |  |  |
| 42 | 0,20 | 0,33 | 97,23 |  |  |  |  |  |  |
| 43 | 0,19 | 0,31 | 97,54 |  |  |  |  |  |  |
| 44 | 0,17 | 0,29 | 97,83 |  |  |  |  |  |  |
| 45 | 0,16 | 0,27 | 98,09 |  |  |  |  |  |  |
| 46 | 0,14 | 0,24 | 98,33 |  |  |  |  |  |  |
| 47 | 0,13 | 0,22 | 98,54 |  |  |  |  |  |  |
| 48 | 0,12 | 0,20 | 98,74 |  |  |  |  |  |  |
| 49 | 0,12 | 0,19 | 98,93 |  |  |  |  |  |  |
| 50 | 0,11 | 0,18 | 99,12 |  |  |  |  |  |  |
| 51 | 0,09 | 0,15 | 99,27 |  |  |  |  |  |  |
| 52 | 0,09 | 0,14 | 99,40 |  |  |  |  |  |  |
| 53 | 0,08 | 0,13 | 99,53 |  |  |  |  |  |  |
| 54 | 0,06 | 0,10 | 99,64 |  |  |  |  |  |  |
| 55 | 0,05 | 0,09 | 99,72 |  |  |  |  |  |  |
| 56 | 0,05 | 0,08 | 99,80 |  |  |  |  |  |  |
| 57 | 0,04 | 0,07 | 99,87 |  |  |  |  |  |  |
| 58 | 0,03 | 0,05 | 99,92 |  |  |  |  |  |  |
| 59 | 0,02 | 0,04 | 99,96 |  |  |  |  |  |  |
| 60 | 0,01 | 0,02 | 99,98 |  |  |  |  |  |  |
| 61 | 0,01 | 0,02 | 100,00 |  |  |  |  |  |  |
| Extraction method: principal component analysis | | | | | | | | |  |

**B.2.5 Factor formation as varimax rotation (9 factors)**

Table B.2.5
Factorization for 61 parameters of LIWC2015 after setting to 9 factors

| Parameter |  | factor loading | | | | | | | | |
| --- | --- | --- | --- | --- | --- | --- | --- | --- | --- | --- |
| LIWC2015 |  | 1 | 2 | 3 | 4 | 5 | 6 | 7 | 8 | 9 |
| *filler* |  | **-0,848** | 0,022 | 0,058 | 0,089 | -0,167 | -0,25 | -0,018 | 0,117 | 0,1 |
| *nonflu* |  | **-0,843** | -0,023 | -0,048 | 0,14 | -0,12 | -0,151 | 0,038 | 0,127 | 0,063 |
| *tentat* |  | **-0,827** | -0,117 | 0,265 | 0,118 | 0,035 | 0,007 | 0,029 | 0,248 | 0,053 |
| *focuspresent* |  | **0,757** | 0,209 | 0,145 | 0,139 | -0,231 | 0,095 | 0,061 | 0,242 | 0,122 |
| *auxverb* |  | **0,75** | -0,017 | 0,004 | 0,372 | -0,066 | 0,049 | 0,014 | 0,169 | 0,088 |
| *verb* |  | **0,727** | 0,087 | 0,109 | 0,449 | -0,127 | 0,156 | 0,051 | 0,314 | 0,077 |
| *prep* |  | **-0,681** | -0,182 | 0,097 | 0,083 | 0,013 | 0,375 | 0,037 | -0,045 | -0,051 |
| *WPS* |  | **-0,578** | -0,256 | -0,072 | 0,115 | -0,115 | 0,202 | 0,283 | 0,083 | -0,4 |
| *Sixltr* |  | **-0,535** | 0,126 | -0,082 | 0,368 | 0,031 | -0,069 | -0,243 | -0,037 | 0,148 |
| *space* |  | **-0,516** | -0,003 | 0,375 | -0,016 | -0,209 | 0,319 | 0,2 | 0,026 | -0,16 |
| *relig* |  | *-0,377* | 0,095 | 0,042 | -0,046 | 0,135 | 0,281 | -0,01 | 0,231 | 0,064 |
| *certain* |  | *0,285* | -0,258 | 0,209 | 0,135 | -0,282 | 0,051 | 0,274 | 0,151 | 0,127 |
| *affiliation* |  | 0,225 | **0,763** | -0,008 | -0,023 | 0,012 | -0,119 | 0,091 | 0,133 | -0,165 |
| *family* |  | 0,269 | **0,756** | -0,251 | 0,051 | -0,034 | 0,064 | -0,091 | -0,138 | 0,016 |
| *sexual* |  | 0,1 | **0,73** | -0,102 | -0,027 | 0,145 | -0,108 | -0,098 | 0,085 | -0,134 |
| *female* |  | -0,1 | **0,596** | -0,165 | 0,067 | 0,109 | 0,164 | 0,123 | -0,063 | 0,055 |
| *conj* |  | 0,37 | **-0,477** | 0,118 | -0,219 | -0,01 | 0,296 | 0,302 | 0,011 | -0,276 |
| *interrog* |  | 0,224 | **-0,428** | 0,105 | -0,047 | 0,361 | 0,282 | 0,215 | 0,097 | 0,036 |
| *cause* |  | 0,21 | **-0,407** | 0,194 | -0,013 | 0,143 | -0,058 | 0,292 | -0,079 | -0,119 |
| *we* |  | 0,138 | *0,386* | 0,005 | 0,053 | -0,101 | -0,291 | 0,126 | 0,086 | -0,147 |
| *swear* |  | -0,041 | *0,385* | 0,022 | 0,086 | -0,203 | 0,128 | 0,024 | -0,08 | 0,086 |
| *power* |  | 0,095 | *0,38* | -0,362 | 0,017 | -0,148 | -0,143 | -0,001 | -0,155 | 0,047 |
| *differ* |  | 0,164 | -0,254 | **0,701** | 0,208 | 0,054 | 0,077 | -0,008 | 0,021 | 0,049 |
| *negate* |  | 0,111 | -0,131 | **0,68** | -0,014 | -0,12 | -0,197 | -0,041 | 0,095 | 0,221 |
| *discrep* |  | 0,311 | -0,248 | **0,616** | 0,073 | 0,005 | 0,28 | 0,038 | 0,424 | 0,104 |
| *reward* |  | 0,392 | 0,257 | **-0,591** | 0,182 | -0,008 | -0,128 | 0,002 | -0,251 | -0,151 |
| *posemo* |  | 0,339 | 0,41 | **-0,553** | 0,032 | 0,323 | -0,27 | 0,081 | -0,132 | -0,105 |
| *adverb* |  | -0,097 | -0,22 | **0,494** | 0,314 | -0,001 | 0,009 | 0,248 | -0,134 | -0,268 |
| *home* |  | 0,044 | 0,326 | **0,403** | -0,029 | 0,002 | 0,214 | -0,041 | -0,126 | 0,008 |
| *they* |  | 0,084 | 0,062 | *-0,396* | 0,13 | -0,181 | 0,229 | 0,32 | -0,045 | 0,265 |
| *feel* |  | 0,268 | -0,101 | *-0,333* | 0,151 | 0,185 | -0,048 | -0,238 | -0,061 | 0,03 |
| *article* |  | 0,032 | -0,113 | -0,24 | **-0,828** | 0,066 | 0,025 | -0,135 | 0,066 | 0,113 |
| *ipron* |  | 0,222 | -0,235 | -0,038 | **-0,693** | 0,015 | -0,004 | 0,078 | 0,026 | -0,05 |
| *focuspast* |  | 0,145 | -0,125 | -0,171 | **0,661** | 0,005 | 0,037 | 0,097 | -0,102 | -0,087 |
| *i* |  | 0,4 | 0,215 | 0,097 | **0,489** | -0,171 | 0,137 | -0,328 | 0,02 | -0,15 |
| *leisure* |  | 0,041 | -0,019 | 0,17 | **-0,459** | -0,093 | 0,259 | 0,272 | -0,313 | 0,27 |
| *achiev* |  | -0,019 | -0,116 | -0,125 | -0,102 | **0,817** | -0,293 | -0,007 | 0,152 | 0,032 |
| *work* |  | -0,189 | -0,072 | -0,054 | -0,034 | **0,685** | -0,196 | -0,056 | 0,354 | 0,005 |
| *compare* |  | -0,117 | -0,221 | 0,227 | 0,174 | **0,68** | 0,207 | 0,094 | -0,104 | -0,109 |
| *adj* |  | 0,348 | 0,203 | -0,169 | 0,195 | **0,602** | 0,106 | -0,061 | -0,346 | -0,079 |
| *number* |  | -0,04 | 0,153 | 0,062 | -0,239 | **0,496** | 0,247 | 0,089 | -0,03 | -0,215 |
| *see* |  | 0,279 | 0,233 | -0,453 | -0,104 | **0,457** | -0,24 | -0,241 | -0,072 | 0,127 |
| *insight* |  | 0,171 | 0,417 | -0,402 | 0,186 | **0,444** | -0,285 | -0,231 | -0,047 | 0,029 |
| *health* |  | -0,086 | 0,201 | 0,102 | 0,027 | *-0,274* | 0,06 | 0,141 | 0,258 | 0,217 |
| *WC* |  | -0,176 | -0,001 | 0,016 | 0,021 | -0,224 | **0,678** | 0,17 | 0,237 | -0,188 |
| *you* |  | 0,009 | 0,046 | 0,063 | 0,074 | 0,051 | **0,566** | 0,11 | -0,109 | 0,222 |
| *motion* |  | 0,075 | -0,127 | 0,132 | 0,01 | -0,055 | **0,462** | -0,128 | 0,066 | 0,11 |
| *money* |  | 0,173 | 0,009 | 0,003 | -0,155 | -0,035 | **0,451** | 0,091 | 0,071 | 0,088 |
| *assent* |  | -0,051 | -0,089 | 0,443 | -0,141 | -0,085 | **-0,443** | 0,072 | -0,115 | 0,39 |
| *risk* |  | 0,061 | -0,008 | 0,018 | -0,015 | 0,145 | 0,019 | **-0,637** | -0,096 | 0,015 |
| *body* |  | 0,032 | 0 | 0,005 | 0,027 | -0,09 | -0,069 | **-0,549** | 0,056 | -0,084 |
| *shehe* |  | 0,219 | -0,124 | -0,418 | 0,455 | 0,047 | -0,173 | **0,475** | 0,109 | 0,074 |
| *hear* |  | 0,079 | 0,003 | 0,127 | 0,078 | 0,022 | 0,096 | *0,358* | 0,205 | -0,189 |
| *focusfuture* |  | -0,016 | 0,003 | 0,125 | -0,051 | 0,161 | 0,127 | 0,143 | **0,708** | 0,137 |
| *quant* |  | -0,121 | -0,234 | 0,077 | 0,416 | 0,087 | -0,217 | 0,31 | **-0,459** | -0,267 |
| *time* |  | -0,173 | -0,202 | 0,219 | -0,232 | -0,055 | 0,032 | 0,281 | **0,422** | -0,205 |
| *friend* |  | -0,047 | 0,22 | 0,363 | -0,093 | 0,138 | 0,063 | 0,29 | *-0,376* | 0,234 |
| *death* |  | -0,045 | -0,121 | 0,044 | -0,21 | -0,011 | 0,076 | 0,065 | 0,112 | **0,673** |
| *negemo* |  | -0,03 | -0,092 | 0,174 | 0,051 | -0,021 | 0,079 | -0,1 | 0,055 | **0,599** |
| *ingest* |  | 0,044 | 0,064 | -0,111 | -0,015 | -0,16 | 0,109 | 0,158 | -0,063 | *0,284* |
| *male* |  | 0,116 | 0,189 | -0,015 | -0,053 | -0,086 | 0,155 | -0,047 | 0,038 | *0,214* |

Note: 7 study participants, explained 56% of total variance; items above 0.4 marked in **bold** and below 0.4 marked in *italics*; extraction method: principal component analysis; values after Varimax rotation with Kaiser normalization.

**B.2.6 Reliability test with cronbach's alpha for the factors of LIWC2015**

Table B.2.6

*Reliability test with cronbach's alpha for the factors of LIWC2015*

| Factor (F_liwc_) | Cronbach´s alpha |
| --- | --- |
| F1_liwc_ | **0.898** |
| F2_liwc_ | **0.779** |
| F3_liwc_ | **0.768** |
| F4_liwc_ | **0.724** |
| F5_liwc_ | **0.766** |
| F6_liwc_ | 0.541 |
| F7_liwc_ | 0.439 |
| F8_liwc_ | 0.489 |
| F9_liwc_ | 0.589 |

Note: Cronbach's alpha > 0.6 **bold**.

**B.2.7 Factor formation of the parameters of LIWC2015**

Table B.2.7

*Factor formation of the parameters of LIWC2015*

| 61 Parameters | Factors | | | | |
| --- | --- | --- | --- | --- | --- |
| LIWC2015 | F1_liwc_ | F2_liwc_ | F3_liwc_ | F4_liwc_ | F5_liwc_ |
| *filler* | -0.848 |  |  |  |  |
| *nonflu* | -0.843 |  |  |  |  |
| *tentat* | -0.827 |  |  |  |  |
| *focuspresent* | 0.757 |  |  |  |  |
| *auxverb* | 0.750 |  |  |  |  |
| *verb* | 0.727 |  |  |  |  |
| *prep* | -0.681 |  |  |  |  |
| *WPS* | -0.578 |  |  |  |  |
| *sixltr* | -0.535 |  |  |  |  |
| *space* | -0.516 |  |  |  |  |
| *affiliation* |  | 0.763 |  |  |  |
| *family* |  | 0.756 |  |  |  |
| *sexual* |  | 0.730 |  |  |  |
| *female* |  | 0.596 |  |  |  |
| *conj* |  | -0.477 |  |  |  |
| *interrog* |  | -0.428 |  |  |  |
| *cause* |  | -0.407 |  |  |  |
| *differ* |  |  | 0.701 |  |  |
| *negate* |  |  | 0.680 |  |  |
| *discrep* |  |  | 0.616 |  |  |
| *reward* |  |  | -0.591 |  |  |
| *posemo* |  |  | -0.553 |  |  |
| *adverb* |  |  | 0.494 |  |  |
| *home* |  |  | 0.403 |  |  |
| *article* |  |  |  | -0.828 |  |
| *ipron* |  |  |  | -0.693 |  |
| *focuspast* |  |  |  | 0.661 |  |
| *i* |  |  |  | 0.489 |  |
| *leisure* |  |  |  | -0.459 |  |
| *achieve* |  |  |  |  | 0.817 |
| *work* |  |  |  |  | 0.685 |
| *compare* |  |  |  |  | 0.680 |
| *adj* |  |  |  |  | 0.602 |
| *number* |  |  |  |  | 0.496 |
| *see* |  |  |  |  | 0.457 |
| *insight* |  |  |  |  | 0.444 |

Allocation of the parameters to the factors (within the factors sorted by descending loading amount); 7 study participants.

**B.3 Exploratory factor analysis of category_v2_scores**

**B.3.1 Principal component analysis (intrinsic value)**

Table B.3.1
Principal component analysis for 52 category_v2_scores according to intrincic value criterion.

|  |  | | | Explained total variance | | |  | | |
| --- | --- | --- | --- | --- | --- | --- | --- | --- | --- |
|  | initial intrinsic value | | | sums of squared  factor loadings for extraction | | | rotated sum of the  squared charges | | |
| compo-nent | total | % of  variance | cumula-ted % | total | % of  variance | cumulated  % | total | % of variance | cumulated% |
| 1 | 24,497 | 47,11 | 47,11 | 24,497 | 47,11 | 47,11 | 17,646 | 33,935 | 33,935 |
| 2 | 12,995 | 24,99 | 72,1 | 12,995 | 24,99 | 72,1 | 9,361 | 18,002 | 51,937 |
| 3 | 7,012 | 13,484 | 85,584 | 7,012 | 13,484 | 85,584 | 9,235 | 17,76 | 69,69 |
| 4 | 3,378 | 6,496 | 92,08 | 3,378 | 6,496 | 92,08 | 7,682 | 14,773 | 84,47 |
| 5 | 2,19 | 4,212 | 96,292 | 2,19 | 4,212 | 96,292 | 6,147 | 11,821 | **96,29** |
| 6 | 0,763 | 1,468 | 97,76 |  |  |  |  |  |  |
| 7 | 0,329 | 0,633 | 98,392 |  |  |  |  |  |  |
| 8 | 0,213 | 0,41 | 98,802 |  |  |  |  |  |  |
| 9 | 0,162 | 0,311 | 99,113 |  |  |  |  |  |  |
| 10 | 0,087 | 0,167 | 99,28 |  |  |  |  |  |  |
| 11 | 0,073 | 0,141 | 99,422 |  |  |  |  |  |  |
| 12 | 0,063 | 0,12 | 99,542 |  |  |  |  |  |  |
| 13 | 0,04 | 0,076 | 99,618 |  |  |  |  |  |  |
| 14 | 0,034 | 0,066 | 99,683 |  |  |  |  |  |  |
| 15 | 0,027 | 0,053 | 99,736 |  |  |  |  |  |  |
| 16 | 0,023 | 0,044 | 99,78 |  |  |  |  |  |  |
| 17 | 0,019 | 0,037 | 99,816 |  |  |  |  |  |  |
| 18 | 0,013 | 0,025 | 99,842 |  |  |  |  |  |  |
| 19 | 0,013 | 0,024 | 99,866 |  |  |  |  |  |  |
| 20 | 0,011 | 0,021 | 99,887 |  |  |  |  |  |  |
| 21 | 0,008 | 0,015 | 99,902 |  |  |  |  |  |  |
| 22 | 0,007 | 0,014 | 99,916 |  |  |  |  |  |  |
| 23 | 0,007 | 0,014 | 99,93 |  |  |  |  |  |  |
| 24 | 0,006 | 0,012 | 99,942 |  |  |  |  |  |  |
| 25 | 0,005 | 0,01 | 99,953 |  |  |  |  |  |  |
| 26 | 0,004 | 0,007 | 99,96 |  |  |  |  |  |  |
| 27 | 0,004 | 0,007 | 99,967 |  |  |  |  |  |  |
| 28 | 0,003 | 0,006 | 99,973 |  |  |  |  |  |  |
| 29 | 0,003 | 0,005 | 99,978 |  |  |  |  |  |  |
| 30 | 0,002 | 0,004 | 99,981 |  |  |  |  |  |  |
| 31 | 0,002 | 0,003 | 99,985 |  |  |  |  |  |  |
| 32 | 0,001 | 0,002 | 99,987 |  |  |  |  |  |  |
| 33 | 0,001 | 0,002 | 99,989 |  |  |  |  |  |  |
| 34 | 0,001 | 0,002 | 99,991 |  |  |  |  |  |  |
| 35 | 0,001 | 0,002 | 99,992 |  |  |  |  |  |  |
| 36 | 0,001 | 0,002 | 99,994 |  |  |  |  |  |  |
| 37 | 0,001 | 0,001 | 99,995 |  |  |  |  |  |  |
| 38 | 0 | 0,001 | 99,996 |  |  |  |  |  |  |
| 39 | 0 | 0,001 | 99,997 |  |  |  |  |  |  |
| 40 | 0 | 0,001 | 99,998 |  |  |  |  |  |  |
| 41 | 0 | 0,001 | 99,998 |  |  |  |  |  |  |
| 42 | 0 | 0,001 | 99,999 |  |  |  |  |  |  |
| 43 | 0 | 0 | 99,999 |  |  |  |  |  |  |
| 44 | 0 | 0 | 99,999 |  |  |  |  |  |  |
| 45 | 0 | 0 | 99,999 |  |  |  |  |  |  |
| 46 | 9,15E-5 | 0 | 100 |  |  |  |  |  |  |
| 47 | 7,45E-05 | 0 | 100 |  |  |  |  |  |  |
| 48 | 5,48E-05 | 0 | 100 |  |  |  |  |  |  |
| 49 | 4,72E-05 | 9,07E-5 | 100 |  |  |  |  |  |  |
| 50 | 2,62E-05 | 5,04E-05 | 100 |  |  |  |  |  |  |
| 51 | 1,76E-05 | 3,38E-05 | 100 |  |  |  |  |  |  |
| 52 | 1,21E-05 | 2,33E-05 | 100 |  |  |  |  |  |  |
| Extraction method: principal component analysis. | | | | | | | | | |

**B.3.2 KMO-test (intrinsic value)**

Table B.3.2
KMO-test as a standard test method for the suitability of the 52 category_v2_scores for exploratory factor analysis.

| **KMO-test** |  |
| --- | --- |
| Measure of sampling suitability according to Kaiser-Meyer-Olkin | 0,884 |

**B.3.3 Parallel analysis**

Table B.3.3
Parallel analysis according to Horn (1965) for 52 parameters of category_v2_scores.

|  | **intrinsic value parallel analysis** |  |
| --- | --- | --- |
|  | **intrinsic value**  **(random numbers)** | **intrinsic value**  **(empirical data)** |
| 1 | 2,603876 | 24,298 |
| 2 | 2,439262 | 13,237 |
| 3 | 2,299351 | 7,008 |
| 4 | 2,181089 | 3,319 |
| 5 | 2,092189 | 2,193 |
| 6 | 2,001413 | ,743 |
| 7 | 1,915992 | ,316 |
| 8 | 1,836087 | ,218 |
| 9 | 1,758357 | ,172 |
| 10 | 1,690237 | ,083 |
| 11 | 1,622119 | ,075 |
| 12 | 1,560705 | ,063 |
| 13 | 1,504781 | ,047 |
| 14 | 1,443448 | ,042 |
| 15 | 1,380717 | ,034 |
| 16 | 1,330125 | ,029 |
| 17 | 1,279426 | ,022 |
| 18 | 1,228141 | ,019 |
| 19 | 1,180874 | ,014 |
| 20 | 1,130557 | ,012 |
| 21 | 1,08675 | ,011 |
| 22 | 1,038268 | ,008 |
| 23 | 0,99749 | ,007 |
| 24 | 0,953208 | ,006 |
| 25 | 0,911163 | ,004 |
| 26 | 0,871346 | ,004 |
| 27 | 0,833026 | ,003 |
| 28 | 0,795001 | ,003 |
| 29 | 0,758013 | ,002 |
| 30 | 0,721951 | ,002 |
| 31 | 0,690017 | ,001 |
| 32 | 0,658606 | ,001 |
| 33 | 0,625522 | ,001 |
| 34 | 0,591631 | ,001 |
| 35 | 0,558333 | ,001 |
| 36 | 0,528252 | ,001 |
| 37 | 0,500897 | ,001 |
| 38 | 0,470462 | ,000 |
| 39 | 0,440865 | ,000 |
| 40 | 0,416308 | ,000 |
| 41 | 0,388629 | ,000 |
| 42 | 0,363502 | ,000 |
| 43 | 0,339183 | ,000 |
| 44 | 0,314123 | ,000 |
| 45 | 0,291014 | ,000 |
| 46 | 0,268582 | 7,864E-05 |
| 47 | 0,244121 | 7,064E-05 |
| 48 | 0,220871 | 5,583E-05 |
| 49 | 0,198543 | 4,379E-05 |
| 50 | 0,175918 | 2,607E-05 |
| 51 | 0,148027 | 1,570E-05 |
| 52 | 0,121633 | 1,081E-05 |

**B.3.4 Factor formation as varimax rotation**

Table B.3.4
Factorization for 52 parameters of the category_v2_scores.

| Parameter | Factor loading | | | | |
| --- | --- | --- | --- | --- | --- |
| *category_v2_scores* | **1** | **2** | **3** | **4** | **5** |
| *enthusiasm* | **0,972** | -0,163 | -0,055 | -0,012 | 0,145 |
| *euphoria* | **0,965** | -0,177 | -0,054 | -0,008 | 0,171 |
| *passion* | **0,963** | -0,178 | -0,056 | -0,01 | 0,179 |
| *cheerfulness* | **0,952** | -0,186 | -0,066 | -0,012 | 0,219 |
| *desire* | **0,945** | -0,19 | -0,07 | -0,016 | 0,242 |
| *amusement* | **0,935** | -0,204 | -0,147 | -0,02 | 0,232 |
| *excitement* | **0,908** | -0,185 | -0,125 | -0,019 | 0,331 |
| *happiness* | **0,903** | -0,214 | -0,196 | -0,025 | 0,29 |
| *delight* | **0,903** | -0,214 | -0,196 | -0,027 | 0,291 |
| *pride* | **0,9** | -0,257 | -0,23 | -0,029 | 0,243 |
| *interest* | **0,898** | -0,248 | -0,124 | -0,019 | 0,329 |
| *badtemper* | **0,887** | -0,071 | 0,346 | 0,244 | -0,039 |
| *outrage* | **0,886** | -0,079 | 0,345 | 0,243 | -0,043 |
| *anger* | **0,867** | -0,05 | 0,39 | 0,25 | -0,05 |
| *agitation* | **0,712** | -0,225 | 0,011 | 0,614 | 0,057 |
| *regret* | **-0,611** | 0,313 | 0,485 | 0,353 | -0,309 |
| *serenity* | -0,27 | **0,854** | -0,339 | -0,245 | -0,091 |
| *compassion* | -0,352 | **0,846** | -0,131 | -0,328 | -0,14 |
| *disgust* | -0,319 | **0,841** | -0,326 | -0,252 | -0,092 |
| *relief* | -0,006 | **0,825** | 0,465 | 0,062 | -0,296 |
| *resentment* | 0,197 | **0,807** | 0,461 | 0,038 | -0,273 |
| *disappointment* | -0,347 | **0,788** | 0,435 | 0,037 | -0,232 |
| *moved* | -0,549 | **0,771** | -0,086 | -0,264 | -0,137 |
| *dejection* | -0,414 | **0,759** | 0,442 | 0,034 | -0,22 |
| *boredom* | -0,576 | **0,754** | -0,016 | -0,242 | -0,177 |
| *sadness* | -0,517 | **0,745** | 0,285 | 0,023 | -0,295 |
| *grief* | 0,615 | **0,643** | -0,373 | -0,217 | -0,07 |
| *contentment* | -0,606 | **0,632** | 0,265 | -0,006 | -0,353 |
| *pleasure* | 0,142 | -0,017 | **-0,863** | -0,116 | 0,322 |
| *impressed* | -0,171 | 0,281 | **-0,848** | -0,172 | 0,182 |
| *surprise* | -0,126 | -0,194 | **-0,825** | -0,102 | 0,372 |
| *frustration* | -0,091 | 0,252 | **0,824** | 0,41 | -0,113 |
| *admiration* | 0,551 | 0,244 | **-0,731** | -0,175 | 0,12 |
| *displeasure* | -0,224 | 0,175 | **0,717** | 0,545 | -0,062 |
| *irritation* | 0,482 | 0,443 | **0,697** | 0,169 | -0,202 |
| *longing* | -0,595 | 0,087 | **-0,679** | -0,294 | 0,139 |
| *hurt* | -0,35 | 0,593 | **0,67** | 0,147 | -0,204 |
| *despair* | -0,321 | 0,114 | **0,666** | 0,601 | -0,116 |
| *suffering* | -0,492 | 0,535 | **0,589** | 0,159 | -0,259 |
| *stress* | 0,089 | -0,111 | 0,165 | **0,967** | 0,004 |
| *shock* | -0,06 | -0,141 | 0,162 | **0,959** | -0,004 |
| *panic* | 0,079 | -0,131 | 0,017 | **0,943** | 0,082 |
| *anxiety* | 0,064 | -0,066 | 0,291 | **0,934** | 0,003 |
| *worry* | 0,281 | -0,183 | 0,053 | **0,916** | 0,039 |
| *agony* | 0,004 | -0,008 | 0,484 | **0,786** | -0,013 |
| *humiliation* | -0,394 | 0,104 | 0,562 | **0,658** | -0,149 |
| *confusion* | 0,185 | -0,239 | -0,233 | 0,01 | **0,913** |
| *guilt* | 0,235 | -0,239 | -0,239 | 0,004 | **0,9** |
| *fear* | 0,364 | -0,226 | -0,222 | 0,013 | **0,866** |
| *nervousness* | 0,315 | -0,162 | -0,163 | 0,061 | **0,864** |
| *highstrung* | 0,431 | -0,24 | -0,224 | -0,001 | **0,827** |
| *loving* | 0,611 | -0,237 | -0,269 | -0,012 | **0,665** |

Note: 7 study participants, explained 96% of total variance. Extraction method: principal component analysis; values after varimax rotation with Kaiser normalization.

**B.3.5 Reliability test with cronbach's alpha for the factors of category_v2_scores**

**Table B.3.5**

*Reliability test with cronbach's alpha for the factors of category_v2_scores.*

| Factor (F_emo_) | Cronbach´s alpha |
| --- | --- |
| F1_emo_ | **0.982** |
| F2_emo_ | **0.964** |
| F3_emo_ | **0.923** |
| F4_emo_ | **0.948** |
| F5_emo_ | **0.977** |

Note: cronbach's alpha > 0.6 **bold**.

**B.3.6 Factor formation of the parameters of category_v2_scores (VocEmoApI)**

**Table B.3.6**

Factor formation of the parameters of category_v2_scores (VocEmoApI*).*

| 52 Parameters | Factors | | | | |
| --- | --- | --- | --- | --- | --- |
| category_v2_scores | F1_emo_ | F2_emo_ | F3_emo_ | F4_emo_ | F5_emo_ |
| *enthusiasm* | 0.972 |  |  |  |  |
| *Euphoria* | 0.965 |  |  |  |  |
| *passion* | 0.963 |  |  |  |  |
| *cheerfulness* | 0.952 |  |  |  |  |
| *desire* | 0.945 |  |  |  |  |
| *amusement* | 0.935 |  |  |  |  |
| *excitement* | 0.908 |  |  |  |  |
| *happiness* | 0.903 |  |  |  |  |
| *delight* | 0.903 |  |  |  |  |
| *pride* | 0.900 |  |  |  |  |
| *interest* | 0.898 |  |  |  |  |
| *badtemper* | 0.887 |  |  |  |  |
| *outrage* | 0.886 |  |  |  |  |
| *anger* | 0.867 |  |  |  |  |
| *agitation* | 0.712 |  |  |  |  |
| *regret* | -0.611 |  |  |  |  |
| *serenity* |  | 0.854 |  |  |  |
| *compassion* |  | 0.846 |  |  |  |
| *disgust* |  | 0.841 |  |  |  |
| *relief* |  | 0.825 |  |  |  |
| *resentment* |  | 0.807 |  |  |  |
| *disappointment* |  | 0.788 |  |  |  |
| *moved* |  | 0.771 |  |  |  |
| *dejection* |  | 0.759 |  |  |  |
| *boredom* |  | 0.754 |  |  |  |
| *sadness* |  | 0.745 |  |  |  |
| *grief* |  | 0.643 |  |  |  |
| *contentment* |  | 0.632 |  |  |  |
| *pleasure* |  |  | -0.863 |  |  |
| *impressed* |  |  | -0.848 |  |  |
| *surprise* |  |  | -0.825 |  |  |
| *frustration* |  |  | 0.824 |  |  |
| *admiration* |  |  | -0.731 |  |  |
| *displeasure* |  |  | 0.717 |  |  |
| *irritation* |  |  | 0.697 |  |  |
| *longing* |  |  | -0.679 |  |  |
| *hurt* |  |  | 0.670 |  |  |
| *despair* |  |  | 0.666 |  |  |
| *suffering* |  |  | 0.589 |  |  |
| *stress* |  |  |  | 0.967 |  |
| *shock* |  |  |  | 0.959 |  |
| *panic* |  |  |  | 0.943 |  |
| *anxiety* |  |  |  | 0.934 |  |
| *worry* |  |  |  | 0.916 |  |
| *agony* |  |  |  | 0.786 |  |
| *humiliation* |  |  |  | 0.658 |  |
| *confusion* |  |  |  |  | 0.913 |
| *guilt* |  |  |  |  | 0.900 |
| *fear* |  |  |  |  | 0.866 |
| *nervousness* |  |  |  |  | 0.864 |
| *highstrung* |  |  |  |  | 0.827 |
| *loving* |  |  |  |  | 0.665 |

Allocation of the parameters to the factors (within the factors sorted by descending loading amount); 7 study participants.

**B.4 Participation statistics of the study patients**

**B.4.1 Participation in the group (picture desing) and in the subsequent private session of TGPR**

Table B.4.1

Participation in the group (picture creation and interview TGPR).

| Patient | Birth year | Gender | Participation | | Special feature |
| --- | --- | --- | --- | --- | --- |
|  |  |  | Picture design | TGPR |  |
| P1 | 1967 | M | 19 | 17 | _ |
| P2 | 1958 | M | 19 | 19 | _ |
| P3 | 1970 | W | 19 | 19 | _ |
| P4 | 1963 | M | 19 | 18 | _ |
| P5 | 1956 | W | 16 | 16 | _ |
| P6 | 1954 | M | 9 (10) | 9 (10) | Moved out in August 2016, but participation as an external on October 29, 2016 |
| P7 | 1960 | W | 19 | - | 'Silent participant'; consent to digital interview recording withdrawn; reliable participation in Image-creation |
| P8 | 1965 | M | 16 | 16 | Missed four weeks because of an internship at the WfbM (Workplace for disabled persons) |

Note: M = male; W = female.

**B.4.2 Participation of patients in the interview-sessions (TGPR) - overview**

Table B.4.2
Participation of the study patients (P1 - P8) in the interview-sessions.

| **I** | **P1** | **P2** | **P3** | **P4** | **P5** | **P6** | **P7** | **P8** |
| --- | --- | --- | --- | --- | --- | --- | --- | --- |
| **1** | 09.05.16 | 09.05.16 | 09.05.16 | 10.05.16 | K | K | `silent | 10.05.16 |
| **2** | 17.05.16 | 19.05.16 | 19.05.16 | 19.05.16 | 19.05.16 | 17.05.16 | participant´ | 19.05.16 |
| **3** | 23.05.16 | 23.05.16 | 23.05.16 | 24.05.16 | 24.05.16 | 24.05.16 |  | 24.05.16 |
| 4 | I canceled | I canceled | I canceled | I canceled | I canceled | I canceled |  | I canceled |
| **5** | 06.06.16 | 06.06.16 | 07.06.16 | 08.06.16 | 08.06.16 | 06.06.16 |  | 08.06.16 |
| 6 | I canceled | I canceled | I canceled | I canceled | I canceled | I canceled |  | I canceled |
| 7 | I canceled | I canceled | I canceled | I canceled | I canceled | I canceled |  | I canceled |
| 8 | I canceled | I canceled | I canceled | I canceled | I canceled | I canceled |  | I canceled |
| **9** | 04.07.16 | 04.07.16 | 05.07.16 | 05.07.16 | 07.07.16 | 04.07.16 |  | P |
| **10** | T defekt | 12.07.16 | 11.07.16 | 12.07.16 | 14.07.16 | 11.07.16 |  | P |
| **11** | 18.07.16 | 18.07.16 | 19.07.16 | 19.07.16 | 19.07.16 | 18.07.16 |  | P |
| **12** | 25.07.16 | 26.07.16 | 25.07.16 | 26.07.16 | 26.07.16 | 26.07.16 |  | P |
| **13** | 02.08.16 | 09.08.16 | K | 02.08.16 | 02.08.16 | 02.08.16 |  | 02.08.16 |
| **14** | 16.08.16 | 16.08.16 | 16.08.16 | 16.08.16 | K | 12.08.16 |  | 16.08.16 |
| **15** | K | 22.08.16 | 22.08.16 | 23.08.16 | 23.08.16 | A |  | 22.08.16 |
| **16** | I refused | 30.08.16 | 30.08.16 | 30.08.16 | 30.08.16 | A |  | 30.08.16 |
| **17** | 05.09.16 | 05.09.16 | 06.09.16 | 06.09.16 | 06.09.16 | A |  | 06.09.16 |
| **18** | 13.09.16 | 12.09.16 | 12.09.16 | 12.09.16 | 16.09.16 | A |  | 12.09.16 |
| **19** | 19.09.16 | K | 19.09.16 | K | K | A |  | 19.09.16 |
| **20** | 26.09.16 | 26.09.16 | 26.09.16 | K | 26.09.16 | A |  | 28.09.16 |
| **21** | 05.10.16 | 04.10.16 | 05.10.16 | 05.10.16 | 04.10.16 | A |  | 04.10.16 |
| 22 | I canceled | I canceled | I canceled | I canceled | I canceled | A |  | I canceled |
| **23** | 17.10.16 | 18.10.16 | 17.10.16 | 18.10.16 | K | A |  | 17.10.16 |
| **24** | 25.10.16 | 24.10.16 | 24.10.16 | 25.10.16 | 25.10.16 | A |  | 24.10.16 |
| **25** | 29.10.16 | 29.10.16 | 29.10.16 | 29.10.16 | 29.10.16 | 29.10.16 |  | 29.10.16 |

Note: A = moving; I = interview; K = sick; P = practical training; T = technique; measurement times (interviews) = **bold**.
